# Prevalence of person-centered maternity care during childbirth and its predictors among women who gave birth in health facilities of East Africa: A systematic review and meta-analysis

**DOI:** 10.1371/journal.pgph.0005909

**Published:** 2026-09-29

**Authors:** Yaregal Admasu Alelign, Chernet Getnet, Alemitu Ayele, Derese Eshetu Debela, Degefa Gomora, Aynamaw Embiale, Yordanos Sintayehu, Neway Ejigu

**Affiliations:** 1 Department of Midwifery, College of Medicine and Health Sciences, Madda Walabu University, Bale Robe, Ethiopia; 2 Department of Biostatistics, School of Public Health, College of Medicine and Health Sciences, Madda Walabu University, Bale Robe, Ethiopia; New Zealand Institute of Public Health, UNITED KINGDOM OF GREAT BRITAIN AND NORTHERN IRELAND

## Abstract

Poor quality care significantly contributes to preventable maternal morbidity and mortality in sub-Saharan Africa, where respectful and responsive childbirth care remains variable. Person-centered maternity care (PCMC) is a crucial element of the WHO maternal and newborn quality-of-care framework; however, region-specific synthesis is lacking. This review aimed to estimate the pooled mean PCMC score and identify its predictors among postpartum women in East Africa. We systematically searched PubMed, Research4Life, ScienceDirect, DOAJ, and Google Scholar for observational studies conducted from 2017 to 2025. Study quality was appraised using the Joanna Briggs Institute critical appraisal checklist. Random-effects meta-analysis using the DerSimonian–Laird estimator was performed, and subgroup and meta-regression analyses were used to explore heterogeneity. Twenty three studies involving 15,325 women were included. The pooled mean PCMC score in East Africa was 63.08% (95% CI: 59.19, 66.97). Domain-specific scores were highest for dignity and respect (78.05%; 95% CI: 74.43–81.67), followed by supportive care (62.59%; 95% CI: 59.44–65.73), and lowest for communication and autonomy (55.29%; 95% CI: 48.66–61.92), demonstrating significant gaps in provider–patient communication and shared decision-making. Lower PCMC scores were associated with nighttime delivery (β = −2.23; 95% CI: −3.35, −1.11), third-trimester antenatal care initiation (β = −4.06; 95% CI: −7.74, −0.38), and rural residence (β = −1.61; 95% CI: −2.60, −0.61), while higher maternal education was positively associated with PCMC. PCMC in East Africa remains suboptimal, predominantly in the communication, autonomy, and supportive care domains. Reinforcing provider communication and shared decision-making skills, improving night-shift staffing and supervision, integrating PCMC indicators into quality monitoring systems, and expanding community-level interventions through task-shifting and community engagement strategies are recommended. Future research should prioritize implementation and intervention studies targeting identified gaps. The protocol has been registered in PROSPERO (CRD42024576229).

## Introduction

The burden of maternal death remains disproportionately high in East Africa. According to World Health Organizations (WHO) estimate in 2020–2023, sub-Saharan Africa contributes to nearly 70% of the global maternal deaths (estimated at 454 maternal deaths/100,000 live births), and many East African countries continue to have maternal mortality ratios exceeding 300 deaths per 100,000 live births [1].

The quality of maternity care often falls short of international standards in underdeveloped regions like East Africa [2,3]. Many women in this region are left vulnerable to mistreatment and neglect during childbirth, discouraging them seeking maternal and child health services in the future, leading to the high maternal death [2–5]. Regardless of advancing maternal healthcare services, significant discrepancies in institutional delivery persists throughout East Africa, specifically among rural and socioeconomically marginalized communities [6]. Poor person-centered maternity care (PCMC)—encompassing experience of disrespect, ineffective communication, and lack of autonomy—is increasingly recognized as a significant barrier to facility-based childbirth, thereby leading to the low utilization of institutional delivery service [2,7]. This illustrates the importance of enhancing PCMC in order to improve quality of care and rise institutional delivery uptake, thereby reducing preventable maternal mortality in the region.

The WHO designated maternal and childhealth care as a key priority to avert these challenge by giving a particular emphasis on PCMC approach in its standards for improving quality of maternal and newborn care framework [8,9]. PCMC is an approach to care that empowers women by prioritizing their unique needs and preferences throughout maternity care provision [8]. It places the woman’s experience at the core of their care, emphasizing respectful and responsive care during childbirth to ensure that each woman feels acknowledged, heard, and valued [7,8].

From the eight designated WHO standards for improving quality of maternal and newborn care, standards 4, 5, and 6 emphasize the three core dimensions of PCMC—**dignity and respect, communication and autonomy, as well as supportive and responsive care** experienced by women during childbirth [9]. **Standard 4** underscores the importance of **communication and autonomy,** ensuring that women receive complete information about their care and are actively involved in all decisions concerning their treatment**. Standard 5** focuses on providing **full respect and preserving the dignity** of all women throughout their stay at the health facility. Finally, **Standard 6** emphasizes the need for **emotional support** that is responsive to each woman’s needs, fostering a sense of empowerment and capability during her childbirth experience.

However, achieving this standard remains challenging in East Africa [2,10]. Many women gave birth in facilities that are understaffed or lack basic medical supplies, and **inefficient health systems**—leaving them without the respectful and supportive standards that PCMC envisions [2,11,12] where this challenge would impose negative maternal and neonatal health outcomes [2,11]. Such negative childbirth experiences make women resistant to return to healthcare facilities for future pregnancies, opting out home births instead where the risks of maternal and neonatal complications are much higher [2,12].

Research on PCMC in East Africa are isolated within individual studies each reporting inconsistent findings with significant variation—ranging from 19.07 out of 39 [13] to 66.4 out of 90 [14], focusing on different healthcare settings and populations with diverse sociocultural background as well as measured with different tool length, making it difficult to get the full figure of PCMC and key predictors in the region. Predictors such as women’s education level, type of health care facility, time and mode of delivery, number and type of providers attending the delivery, and women’s socioeconomic background are thought to affect the provision of PCMC [4,10,15–18]. Growing evidence regarding PCMC remains fragmented within individual studies, with no prior comprehensive synthesis examining the pooled PCMC estimate, subdomains, and predictors across East Africa, despite increasing institutional delivery in the region.

Therefore, this **systematic review and meta-analysis** aimed to assess the **extent of** PCMC and its domains—**dignity and respect, communication and autonomy, and supportive care** and identify **key predictors** that affect its provision in East Africa. The study provides valuable information for healthcare policymakers and health care professionals the current status of PCMC in East Africa and design effective implementations to enhance the provision of PCMC in the region.

## Methods

### Study design

This systematic review and meta-analysis was conducted to estimate the pooled mean score of PCMC and its predictors in East Africa. The design of this review adhered to the Preferred Reporting Items for Systematic Reviews and Meta-Analyses (PRISMA) statement [19] and Meta-Analysis of Observational Studies in Epidemiology (MOOSE) guidelines [20] for literature searching method, study selection, data extraction, and reporting. The PRISMA statement is available in the S1 Checklist.

The review protocol was registered in the International Prospective Register of Systematic Reviews (PROSPERO) database with CRD42024576229 and available in: https://www.crd.york.ac.uk/prospero/display_record.php?RecordID=576229.

### Search strategy

A comprehensive search of studies was conducted from inception to December 24, 2025 using databases such as PubMed, Research4Life, ScienceDirect, and Directory of Open Access Journals (DOAJ). Additionally, we searched articles missed on databases using Google Scholar. Moreover, we performed a manual search using the reference list of included articles to capture relevant articles. Search terms were conducted using condition, context, population (CoCoPop) technique. The following key terms combined with Medical Subject Heading (MeSH) terms were employed for PubMed search: “Person-Centered Care”, “Person-Centered Maternity Care”, “Patient-Centered Care”, “Woman Centered Care”, Client Centered Care”, “Maternity Care”, “Childbirth Experience”, “Childbirth”, and “Africa, Eastern” using Boolean operators “AND” and “OR”. Filters such as **female, humans, and publication date from** 2017/1/1 to 2025/12/24 were applied. Otherwise, the search was conducted without language restrictions. Details of databases search are available in the S1 Table.

### Eligibility criteria

To establish the eligibility criteria, we employed the CoCoPop principle.

#### Inclusion criteria.

**Study design:** All observational studies that reported the prevalence and/or at least one adjusted predictor of PCMC were included.

**Study area and period:** Studies conducted in health facilities of East Africa between 2017 and 24 December 2025 were considered. The restriction to 2017 onward was applied because the validated 30-item PCMC scale was first developed and operationalized in 2017, and studies conducted before this period used non-comparable measures.

**Publication status and language:** Both published and unpublished studies without language restrictions were included.

**Study setting:** Both community and institution-based studies were considered.

**Condition:** PCMC during childbirth

**Context:** East Africa

**Population:** Postpartum women

#### Exclusion criteria.

We excluded case reports, conferences abstracts, editorials, qualitative studies, and systematic reviews. We also excluded articles that are not fully accessible after contacting the primary/corresponding author since assessing methodological quality in the absence of full text was not feasible. Studies lacking sufficient information to derive the required effect estimates were excluded from the corresponding quantitative synthesis if the missing data could not be obtained or calculated.

### Study selection

This review included published and unpublished studies on PCMC and its predictors among postpartum women in East Africa. Electronic search was done on databases such as PubMed, ScienceDirect, Reseach4Life, Directory of Open Access Journals (DOAJ), and Google Scholar. All identified articles were imported into Mendeley Reference Manager and checked for duplication. After removing duplicate articles, eight authors (YAA, NE, CG, AA, DED, AE, DG, and YS) screened all potentially eligible articles independently by title and abstract. Their bibliographies were then manually checked to ensure that no potentially relevant studies were missed. Two authors (YAA and NE) independently screened full text articles for eligibility and any disagreement raised was resolved by a third reviewer (CG) and evidence-based discussion. The complete list of records identified through the literature search, including studies screened at full text and reasons for exclusion is provided in the S2 Table.

### Data extraction

Data were extracted using Microsoft Excel 21. Two authors (YAA and NE) separately extracted the important data using a pre-settled, piloted, and standard data extraction format which comprised the author’s name, publication year, study country, study design, study setting, sampling technique, sample size, response rate, mean of PCMC and each sub-scale, standard deviation (SD) of PCMC and each sub-scale, and predictors of PCMC with beta coefficient (β) along with their 95% Confidence Intervals (CIs). For studies with incomplete or insufficiently reported outcome data, we extracted relevant information from tables, figures, supplementary materials, or related reports whenever possible. Studies lacking sufficient data to derive the required effect estimates were excluded from the corresponding quantitative synthesis. No statistical imputation was performed for missing data. Details of full extracted datasets for all studies included in the systematic review and meta-analysis are available in the S1 Data.

### Risk of bias (quality) assessment

We assessed methodological quality using the Joanna Briggs Institute (JBI) Critical Appraisal Checklist for Prevalence Studies [21]. Two authors (YAA and NE) independently evaluated the quality of included studies. The checklist comprises of nine components: (1) appropriateness of the sampling frame, (2) appropriateness of sampling method, (3) adequacy of sample size, (4) description of study subjects and setting, (5) coverage of the identified sample, (6) validity of condition identification, (7) reliability of measurement, (8) appropriateness of statistical analysis, and (9) adequacy of response rate. Each of the nine domains was scored as “Yes” (1) or “No/Unclear/ Not applicable” (0), yielding a total score ranging from 0 to 9. Studies scoring 7–9 were considered low risk of bias, 4–6 moderate risk, and 0–3 high risk of bias [22,23]. Discrepancy between authors that arise during quality assessment was resolved based on evidence-based discussion. Detailed item-level judgments and overall risk-of-bias classifications for each included study are provided in the S3 Table.

### Measurement of outcome of interest

The main outcome of interest for this systematic review and meta analysis was the pooled mean PCMC score (including its subscale) and its predictors among women who gave birth in health facilities of East Africa. PCMC was measured using the validated and standardized 30-item PCMC scale, which is the composite score of the three domains: dignity and respect (6-items), communication and autonomy (9-items), and supportive care (15-items) [7] and the 13-item PCMC scale [24]. For Multicenter studies that report separate findings by study site, we treated each site as an independent study in our meta-analysis because of the distinct study populations across study sites [14,25]. This helps to avoid unit-of-analysis errors and is in accordance with the Cochrane handbook of systematic reviews of interventions [26]. Before the actual analysis, raw summary measures, such as the mean and standard deviation (SD) for PCMC and each subscale were rescaled to a standardized scale (0–100) by dividing the raw score by the maximum possible score multiplied by 100 (standardized mean score = [raw score/maximum possible score] * 100) to ensure comparability and interpretability across studies. The pooled mean PCMC score and its subscale were reported by pooled mean. Predictors of PCMC during childbirth were estimated using the pooled beta coefficients (β) with their corresponding 95% CIs. Predictors reported at least in two primary studies were selected to explore their association with PCMC during childbirth. Predictors assessed for this review were residence, educational level of women, presence of ANC follow-up, timing of ANC follow-up initiation, and time of delivery.

### Statistical methods and analysis

Before the actual analysis, all the necessary data were extracted using Microsoft Excel 21 from original studies and imported into R 4.5.2 statistical software for further analyses. Heterogeneity among studies was assessed by Cochran’s Q test and Higgins I^2^, where I^2^ values of 25%, 50%, and 75% indicate low, moderate, and high heterogeneity, respectively [27]. The statistical significance of heterogeneity was set at *p* < 0.05.

Pooled estimates of PCMC and its domains were derived via random effects meta-analysis with DerSimonian–Laird weights in order to account for anticipated heterogeneity such as variation in study country, populations, settings, and measurement tool. This model offers more conservative estimates in varied situations by accounting for both within- and between-study heterogeneity. To improve robustness, we performed additional analyses, such as subgroup analyses (by country, publication year, study setting, sample size, sampling technique, and measurement scale) and meta-regression using study-level covariates to further investigate sources of heterogeneity, since the DerSimonian–Laird estimator may underestimate between-study variance (τ²) under significant heterogeneity.

The pooled β for predictors were derived via both common effect and random-effects model depending on the observed heterogeneity. The study findings are presented via forest plots. Statistical significance was set at *p* < 0.05.

#### Publication bias and sensitivity analysis.

Publication bias or small-study effects was evaluated using the visual funnel plot and the objective Egger’s test. A sensitivity analysis using the trim-and-fill method was carried out to adjust for publication bias. A leave-one-out sensitivity analysis was also conducted to identify the effect of a single study on the overall pooled result.

#### Sub-group analysis.

Sub-group analyses was conducted by study country, publication year, study setting, sampling technique, measurement scale, and sample size.

#### Meta regression.

To explore heterogeneity, we also conducted meta-regression analyses based on the following covariates: study country, publication year, study setting, sampling technique, and sample size.

## Results

### Study selection

This review included published and unpublished studies on PCMC during childbirth and its predictors among postpartum women in East Africa. Electronic search was done on PubMed, ScienceDirect, Reseach4Life, DOAJ, and Google Scholar. The initial search from these databases identified a total of 3,789 articles. After removing 190 duplicates, 3,599 articles remained. Subsequently, 3,153 and 416 articles were eliminated upon reviewing their titles and abstracts, respectively. Ultimately, 30 full-text articles were assessed and evaluated against the predefined inclusion criteria. Of these, one was excluded due to a lack of full text access [28], four were excluded because of outcome of interest not reported [29–32], one excluded due to study design and different effect size measure [33], and one article was excluded due to different outcome of interest [34]. Consequently, 23 full-text articles were deemed eligible for inclusion in the final quantitative synthesis (Fig 1).

**Fig 1:**
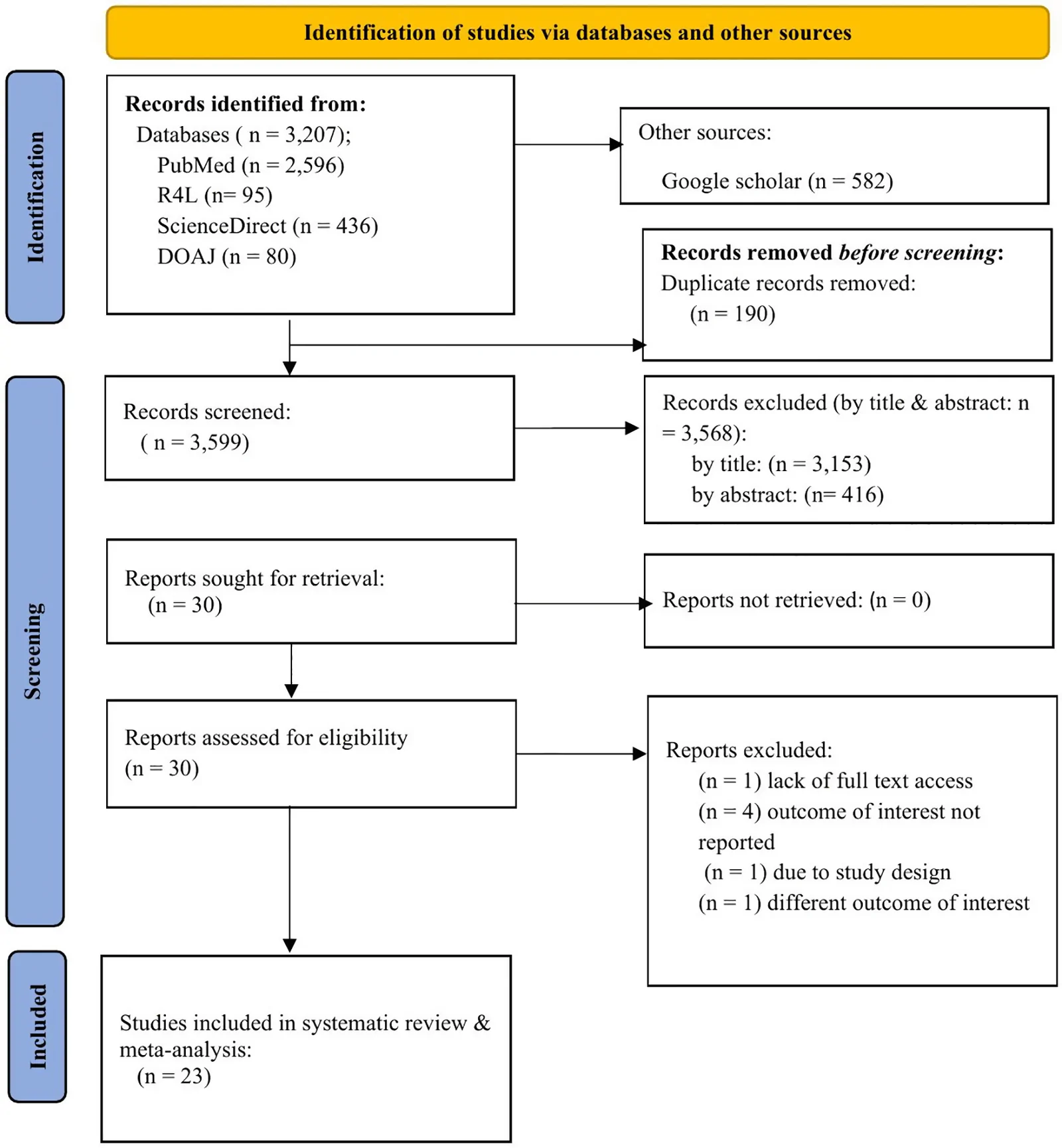
PRISMA flow diagram of the study selection process for a systematic review and meta-analysis of person-centered maternity care during childbirth in East Africa.

Records were identified through PubMed, Research4Life, ScienceDirect, DOAJ, and Google Scholar. After removing duplicates and screening of titles and abstracts, full text articles were assessed for eligibility based predefined inclusion criteria. A total of 23 studies were included in the final systematic review and meta-analysis. The literature search period incorporates studies reported up to December 24, 2025.

### Characteristics of included studies

Twenty three [23] studies were included in this systematic review and meta-analysis. All studies were reported in English language, although we extend our search strategy without language restrictions. Of the studies included in this review, 12 were conducted in Ethiopia [10,13,15–18,35–40] while 11 in Kenya [4,7,14,24,25,41–43]. All studies were conducted by a cross-sectional study design. There were a total of 15,325 study participants among included studies, ranging from 310 [17] to 1575 [13]. The response rate ranges from 80.9% (14) to 100% [39]. The rescaled mean PCMC ranges from 47.00 [35] to 69.5 in Urban Kenya [14] (Table 1).

**Table 1:** Descriptive summary of included studies in a systematic review and meta-analysis of person-centered maternity care in East Africa (n = 23).

Author, Year	Study country	Study design	Study setting	Response rate (%)	Sample size	Raw mean PCMC score	Rescaled mean PCMC score
Afulani et al., 2017 [7]	Kenya	Cross-sectional	Institution based	99.00	1387	59.8	66.44
Afulani et al., (Urban) 2019 [25]	Kenya	Cross-sectional	Institution based	99.81	530	60.2	66.89
Afulani et al., (Rural) 2019 [25]	Kenya	Cross-sectional	Mixed	98.1%	877	59.5	66.11
Alelign et al., 2024 [10]	Ethiopia	Cross-sectional	Institution based	98.19	380	56.83	63.14
Dagnaw et al., 2022 [36]	Ethiopia	Cross-sectional	Institution based	97.40	369	57.6	64.00
Dagnaw et al., 2020 [17]	Ethiopia	Cross-sectional	Community based	97.80	310	58	64.44
Getahun et al., 2022 [16]	Ethiopia	Cross-sectional	Institution based	97.10	564	56	62.22
Oluoch et al., 2021 [41]	Kenya	Cross-sectional	Community based	96.00	307	58.2	64.67
Tarekegne, Giru and Mekonnen., 2022 [18]	Ethiopia	Cross-sectional	Institution based	99.20	384	59.2	65.78
Kabale et al., 2024 [15]	Ethiopia	Cross-sectional	Institution based	98.77	565	60.2	66.89
Nakphong et al., 2024 [42]	Kenya	Cross-sectional	Institution based	95.07	1138	60.02	66.69
Dagnaw, Bizuneh and Kassie., 2024 [35]	Ethiopia	Cross-sectional	Institution based	98.50	763	42.3	47.00
Afulani et al., 2019 [24]	Kenya	Cross-sectional	Institution-based	99.58	1419	24.16	61.95
Stierman et al., 2023 [13]	Ethiopia	Cross-sectional	Community-based	100	1575	19.07	48.90
Kiti et al., 2022 [43]	Kenya	Cross-sectional	Institution-based	82.22	865	24.2	62.05
Afulani et al., 2018 [4]	Kenya	Cross-sectional	Institution-based	98.09	877	59	65.56
Morke 2025 [39]	Ethiopia	Cross-sectional	Institution-based	100%	381	35.03	47
Fata 2022 [40]	Ethiopia	Cross-sectional	Institution-based	99.4%	568	57.2	63.5
Gebreyesus et al., 2025 [38]	Ethiopia	Cross-sectional	Institution-based	98.1%	412	61.29	68.1
Jiwani et al (Lusaka)., 2025 [14]	Kenya	Cross-sectional	Institution-based	88.4%	436	51.4	57.1
Jiwani et al (Ougadougou)., 2025 [14]	Kenya	Cross-sectional	Institution-based	80.9%	401	66.4	73.8
Jiwani et al (Nairobi)., 2025 [14]	Kenya	Cross-sectional	Institution-based	93.4%	412	62.55	69.5
Tassie et al., 2025 [37]	Ethiopia	Cross-sectional	Institution-based	98.8%	405	58.62	69.2

PCMC = person-centered maternity care.

Risk-of-bias assessment using the JBI critical appraisal checklist revealed that all included studies were judged to be low risk of bias (strong quality of evidence). Details of risk-of-bias judgments and domain-level assessments of included studies are available in S3 Table.

Among the domains of PCMC, the highest mean score was observed in the dignity and respect domain [18], while the lowest score was observed in the communication and autonomy domain [35] (Table 2).

**Table 2:** Descriptive summary of raw and standardized mean person-centered maternity care subscale scores in East Africa (n = 20).

Author, Year	Study country	Raw mean DR	Rescaled mean DR	Raw mean CA	Rescaled mean CA	Raw mean SC	Rescaled mean SC
Afulani et al., 2017 [7]	Kenya	14.8	82.22	14.4	53.33	30.5	67.78
Afulani et al., (Urban) 2019 [25]	Kenya	14.4	80.00	15.1	55.93	30.4	67.56
Afulani et al., (Rural) 2019 [25]	Kenya	15.1	83.89	13.9	51.48	30.5	67.78
Alelign et al., 2024 [10]	Ethiopia	15.08	83.78	14.42	53.41	27.33	60.73
Dagnaw et al., 2022 [36]	Ethiopia	14.7	81.67	15.2	56.30	27.7	61.56
Dagnaw et al., 2020 [17]	Ethiopia	14.79	82.17	15.35	56.85	27.87	61.93
Getahun et al., 2022 [16]	Ethiopia	14.6	81.11	15.5	57.41	25.9	57.56
Oluoch et al., 2021 [41]	Kenya	14.7	81.67	15.74	58.30	27.76	61.69
Tarekegne, Giru and Mekonnen., 2022 [18]	Ethiopia	17.7	98.33	14.62	54.15	28.84	64.09
Kabale et al., 2024 [15]	Ethiopia	10.63	59.06	19.85	73.52	26.22	58.27
Nakphong et al., 2024 [42]	Kenya	13.92	77.34	15.99	59.24	30.11	66.90
Dagnaw, Bizuneh and Kassie., 2024 [35]	Ethiopia	13.71	76.17	4.89	18.11	23.72	52.71
Afulani et al., 2018 [4]	Kenya	12.7	70.56	16.5	61.11	29.8	66.22
Morke et al., 2025 [39]	Ethiopia	6.75	56.4	10.99	57.2	24.04	45.4
Fata 2025 [40]	Ethiopia	13.73	76.2	15.78	58.4	27.69	64.5
Gebreyesus et al., 2025 [38]	Ethiopia	14.51	80.6	16.49	61.1	30.29	67.3
Jiwani et al (Lusaka) ., 2025 [14]	Kenya	13.23	73.5	12.85	47.6	25.34	56.3
Jiwani et al (Ougadougou) ., 2025 [14]	Kenya	14.99	83.3	15.82	58.6	35.64	79.2
Jiwani et al (Nairobi)., 2025 [14]	Kenya	15.17	84.3	17.06	63.2	30.33	67.4
Tassie et al., 2025 [37]	Ethiopia	14	73.35	15.6	50.69	39.7	55.88

DR = dignity and respect, CA = communication and autonomy, SC = supportive care.

### The pooled mean scores of PCMC and its domains in East Africa

The pooled mean PCMC score across 23 studies (n = 15,325) was 63.08% (95% CI: 59.19, 66.97) under a random‑effects model, compared to 59.74 (95% CI: 59.54, 59.94) under a common‑effect model (Fig 2). Given substantial heterogeneity (*I*^2^ = 99.7%, *p* < 0.001%), the random‑effects estimate is considered more appropriate. Hence, the pooled estimate should be interpreted cautiously as a broad regional summary rather than an exact estimate of PCMC in all East African contexts. The high heterogeneity likely reflects variation in study populations, health system context, and facility characteristics.

**Fig 2:**
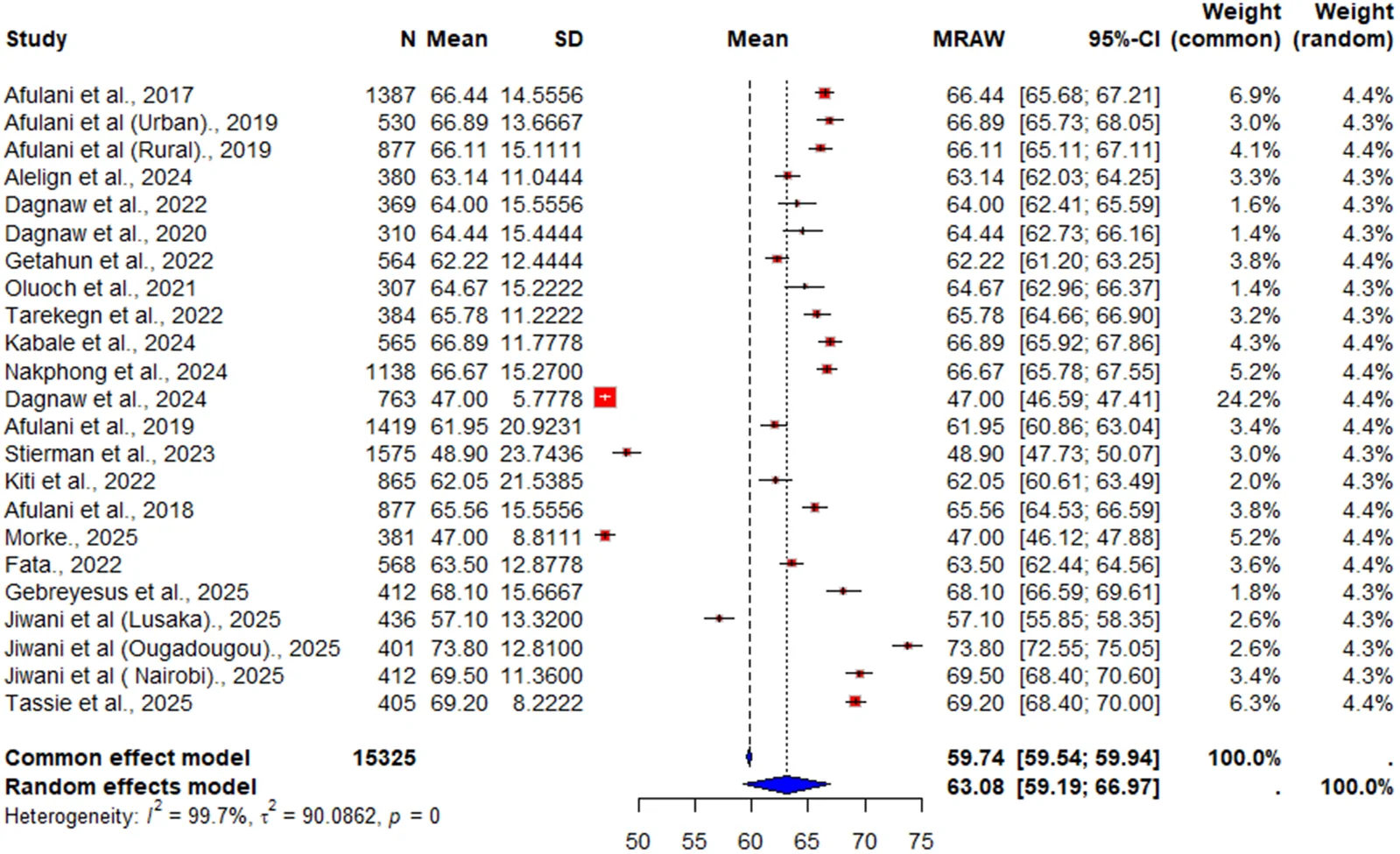
Forest plot showing the pooled mean score of person-centered maternity care in East Africa.

The pooled mean scores of PCMC domains were 78.05% (95% CI: 74.43, 81.67) for dignity and respect (Fig 3), 55.29% (95% CI: 48.66, 61.92) for communication and autonomy (Fig 4), and 62.59% (95% CI: 59.44, 65.73) for supportive care (Fig 5), respectively.

**Fig 3:**
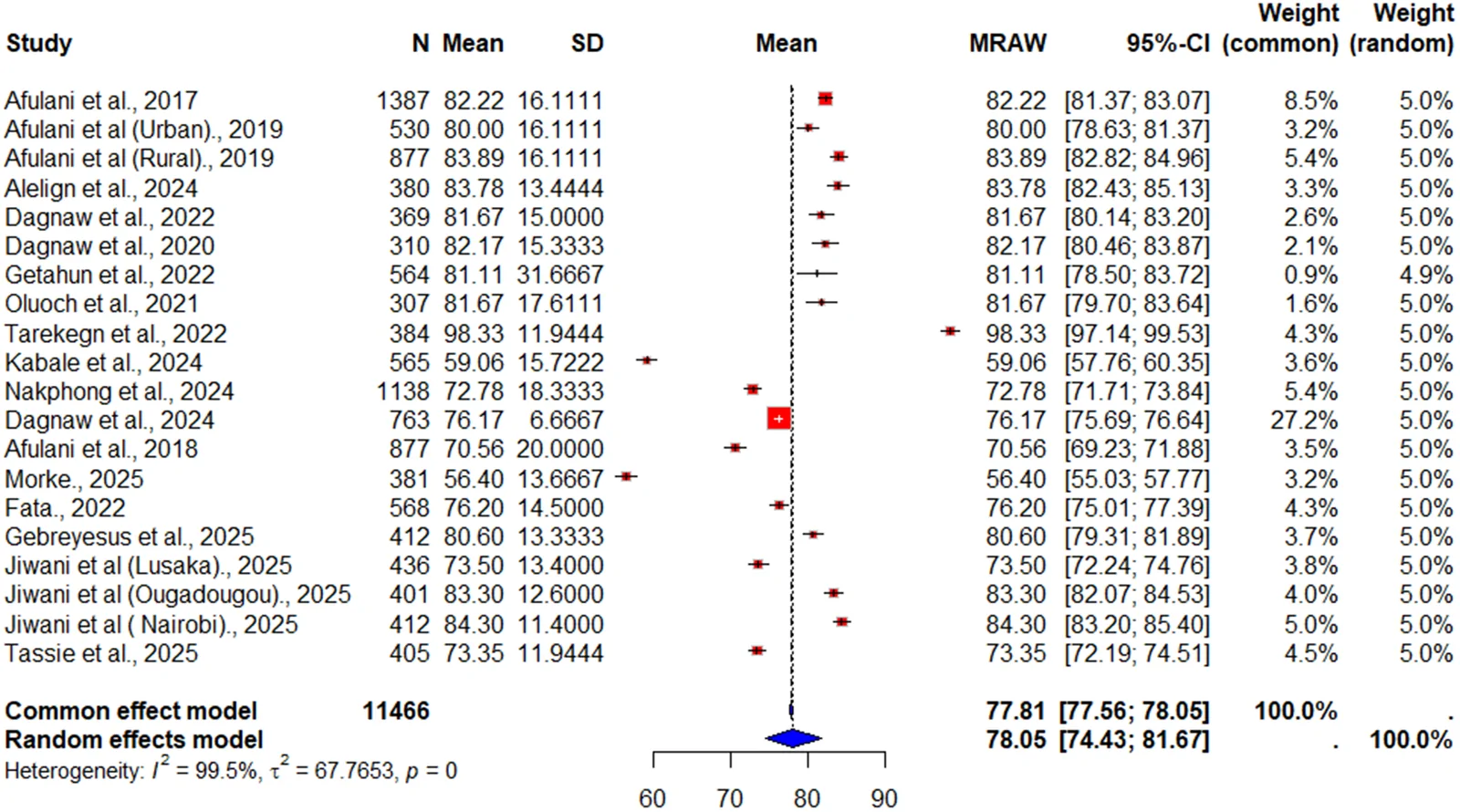
Forest plot showing the pooled mean estimate of the dignity and respect subscale of person-centered maternity care in East Africa.

**Fig 4:**
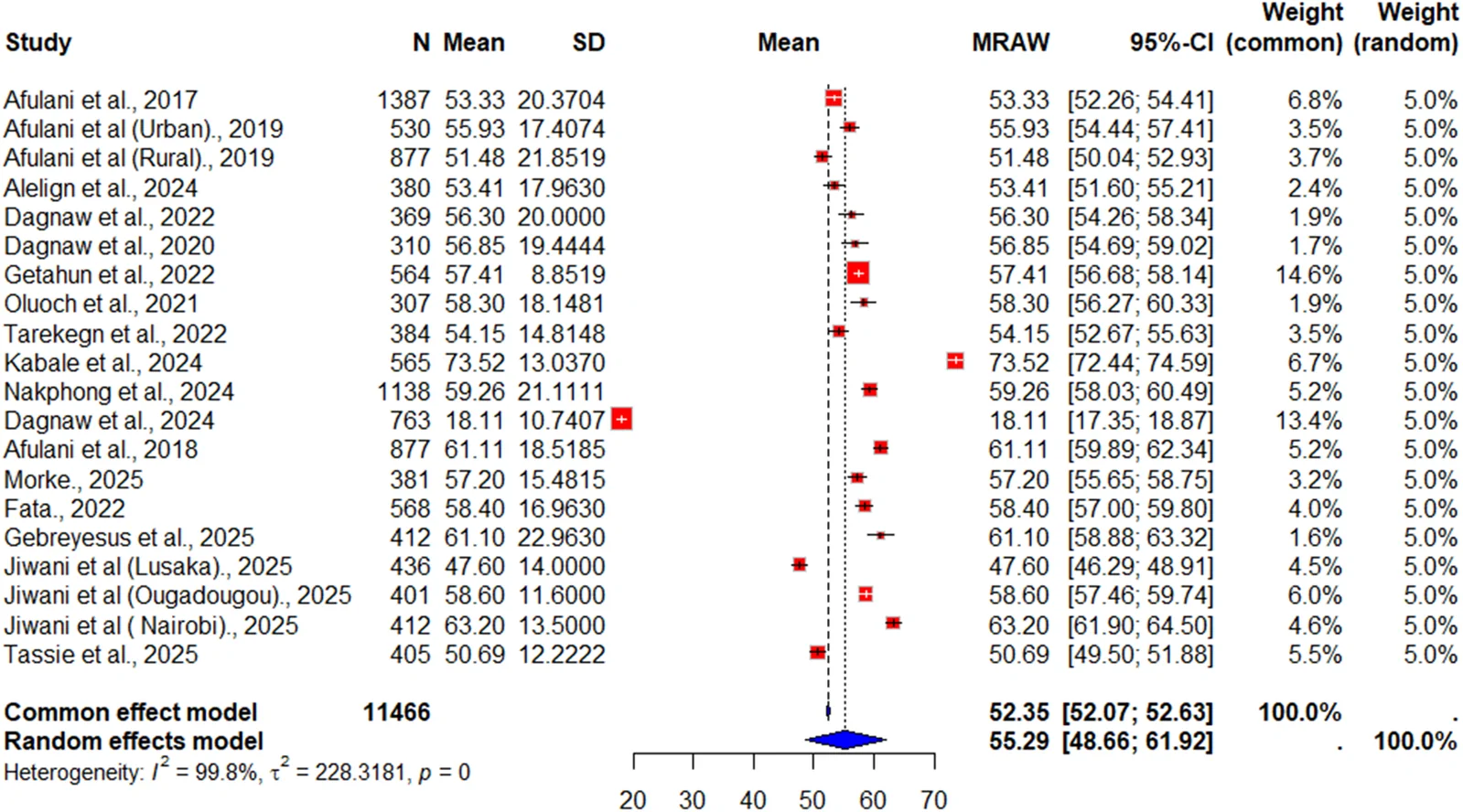
Forest plot of the pooled mean estimate of the communication and autonomy subscale of person-centered maternity care in East Africa.

**Fig 5:**
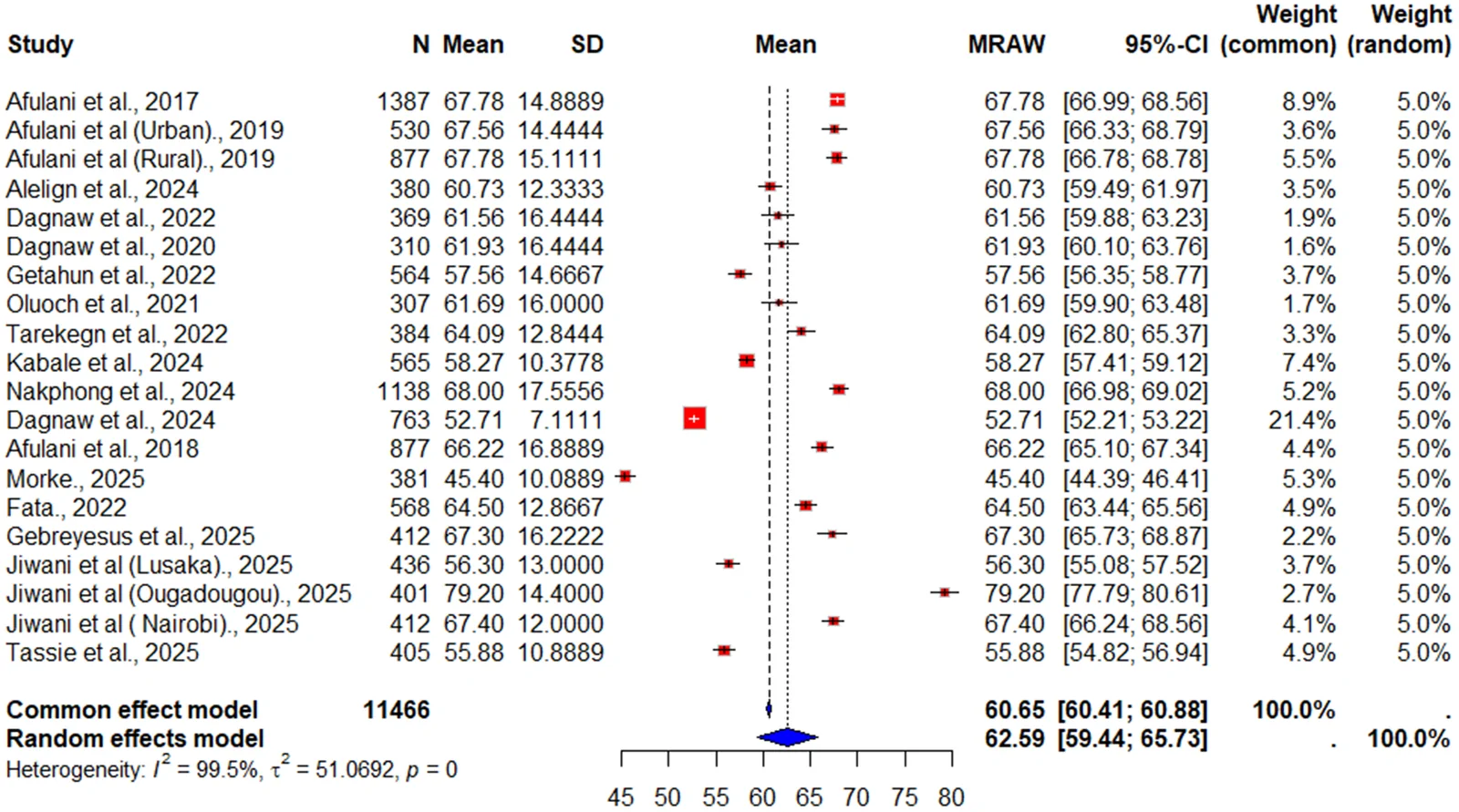
Forest plot showing the pooled mean estimate of the supportive care subscale of person-centered maternity care in East Africa.

### Publication bias and sensitivity analysis

The visual funnel plot showed asymmetry distribution of PCMC studies, suggesting potential publication bias (Fig 6). The objective Egger’s test result revealed a *p*-value of 0.0009 and an intercept of 44.4, suggesting small study effects. In conclusion, the asymmetry in the visual funnel plot and the significant **p-value (0.0009)** in Egger’s test, combined with the **intercept (44.4)**, supports the presence of potential publication bias.

**Fig 6:**
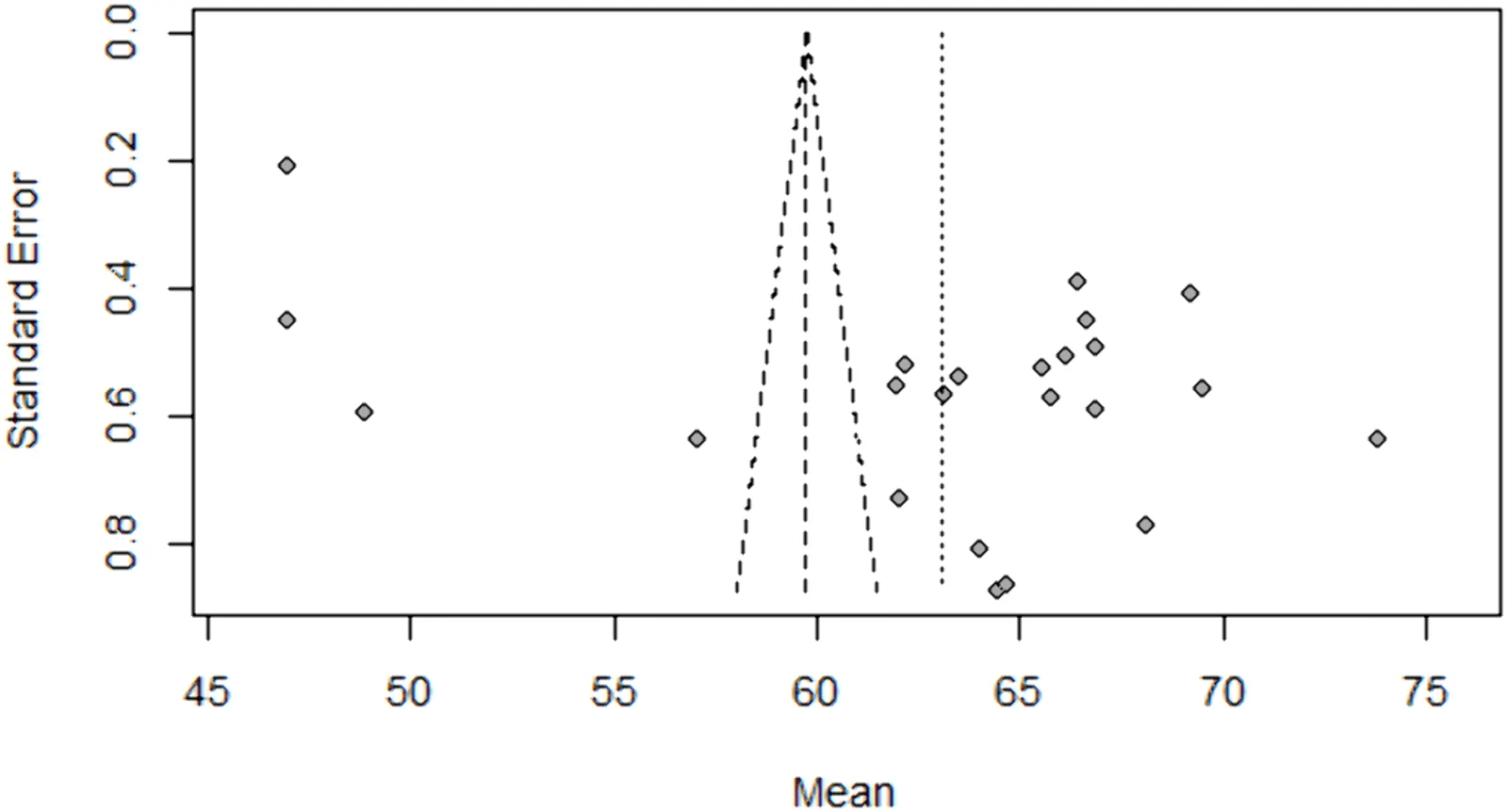
Funnel plot assessing small study effects for the pooled mean estimate of person-centered maternity care during childbirth in East Africa.

A sensitivity analysis using trim-and-fill method revealed the overall pooled mean PCMC score of 55.38% (95% CI: 51.21, 59.55) (Fig 7). This is a slightly more conservative estimate compared to the pre-trim-and-fill analysis. The addition of eleven hypothetical studies suggests small study effects, as the adjusted pooled estimate is lower than the original estimate. The adjusted funnel plot after trim-and-fill analysis demonstrated enhanced symmetry (Fig 8).

**Fig 7:**
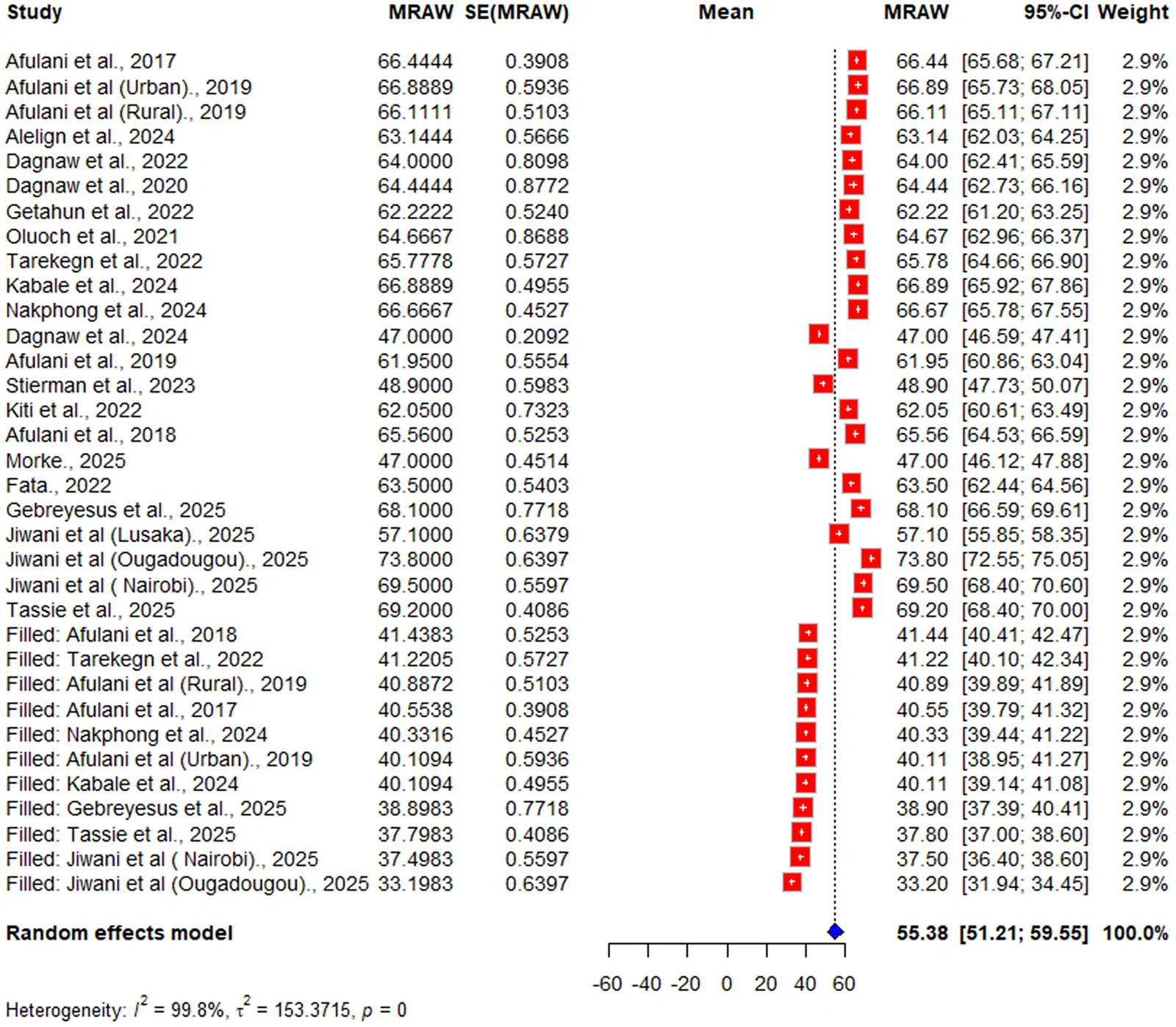
Forest plot assessing the pooled mean estimate of person-centered maternity care during childbirth in East Africa after trim-and-fill analysis.

**Fig 8:**
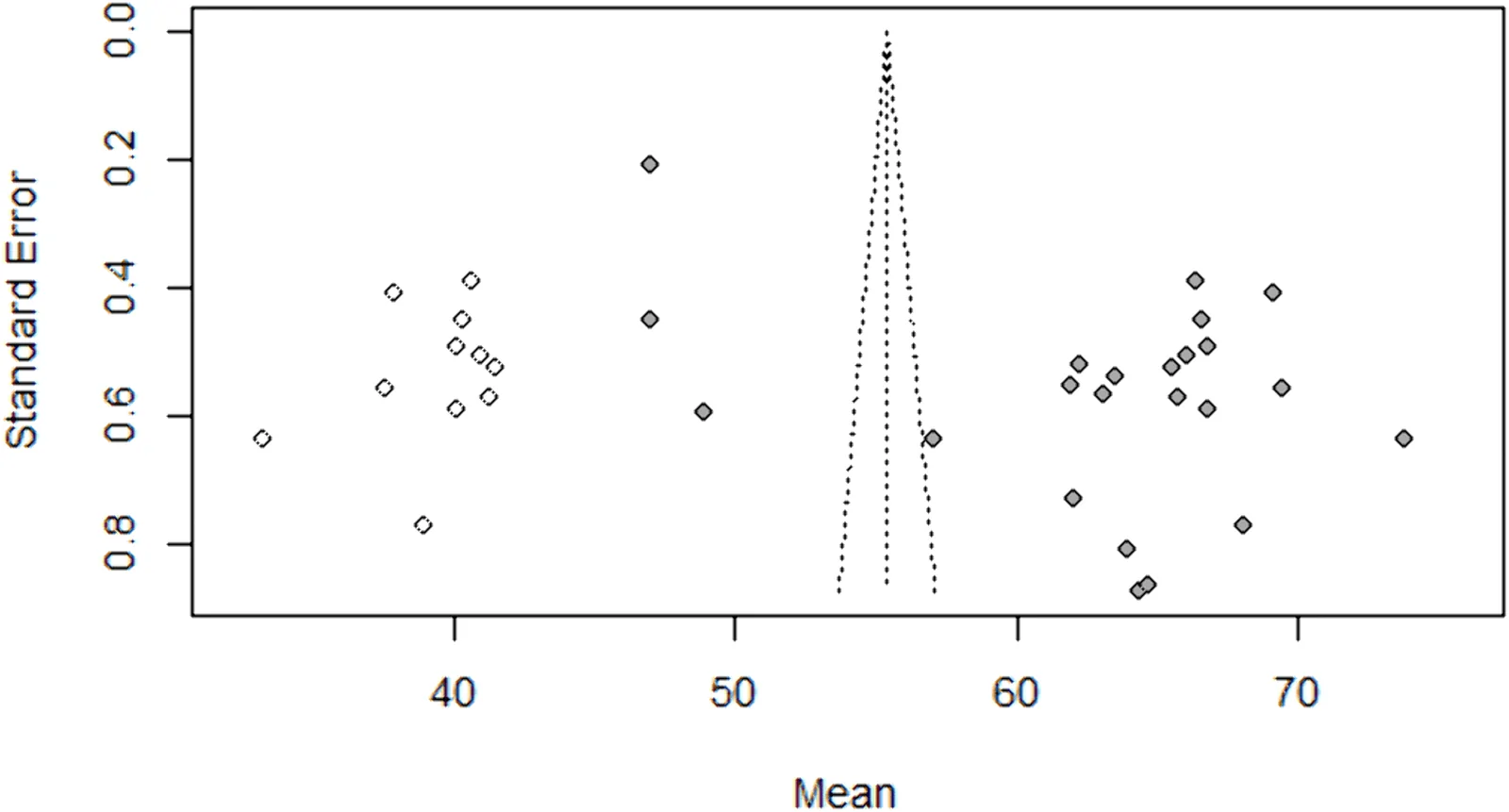
Funnel plot assessing small study effects for the pooled mean score of person-centered maternity care during childbirth in East Africa after trim-and-fill adjustment.

The plot shows individual study estimates against their standard errors. The vertical dashed line represents the pooled mean estimate and the diagonal lines indicate the pseudo 95% CIs. Eleven studies were added using the trim-and-fill method to improve symmetry of funnel plot.

A leave-one-out sensitivity analyses using random-effects meta-analysis model demonstrated that **no single study drastically changes the pooled mean estimate** of PCMC in East Africa, indicating the robustness of the result to the exclusion of any single study, with pooled mean estimate ranging from 62.59 [14] to 63.81 [35,39]. Most iterations showed high heterogeneity (*I*^2^ = 99.7%) and a high tau^2^ value (92.23%), indicating substantial variability across studies. However, excluding Dagnaw et al [35] demonstrated a slightly higher pooled estimate of PCMC 63.81% (95% CI: 61.08, 66.54) with narrower CI and a drop in heterogeneity (τ^2^ = 42.31%), suggesting increased consistency among the remaining studies (Table 3).

**Table 3:** Leave-one-out sensitivity analyses of pooled mean person-centered maternity care scorein East Africa.

Study excluded	K (remaining studies)	Pooled mean PCMC score	95% CI	τ²	I² (%)
None (overall model)	23	63.08	(59.19, 66.97)	92.1	99.7
Afulani et al., 2017	22	62.93	(58.87, 66.98)	93.23	99.7
Afulani et al., (Urban), 2019	22	62.90	(58.90, 66.91)	91.24	99.7
Afulani et al., (Rural), 2019	22	62.94	(58.91, 66.97)	92.22	99.7
Afulani et al., 2018	22	62.97	(58.94, 67.00)	92.72	99.7
Afulani et al., 2019	22	63.13	(59.08, 67.18)	93.63	99.7
Alelign et al., 2024	22	63.08	(59.03, 67.12)	93.23	99.7
Dagnaw et al., 2020	22	63.02	(59.02, 67.02)	91.24	99.7
Dagnaw et al., 2022	22	63.04	(59.03, 67.04)	91.51	99.7
Dagnaw et al., 2024	22	63.81	(61.08, 66.54)	42.31	99.3
Getahun et al., 2022	22	63.12	(59.06, 67.18)	94.01	99.7
Oluoch et al., 2021	22	63.01	(59.01, 67.01)	91.23	99.7
Tarekegn et al., 2022	22	62.96	(58.94, 66.98)	92.22	99.7
Kabale et al., 2024	22	62.91	(58.89, 66.93)	92.14	99.7
Nakphong et al., 2024	22	62.92	(58.89, 66.95)	92.69	99.7
Stierman et al., 2023	22	63.72	(59.77, 67.68)	89.24	99.7
Kiti et al., 2022	22	63.13	(59.11, 67.15)	92.11	99.7
Morke., 2025	22	63.81	(59.94, 67.68)	85.40	99.7
Fata., 2022	22	63.06	(59.01, 67.11)	93.43	99.7
Gebreyesus et al., 2025	22	62.85	(58.87, 66.84)	90.60	99.7
Jiwani et al. (Lusaka), 2025	22	63.35	(59.32, 67.38)	92.71	99.7
Jiwani et al., (Ouagadougou), 2025	22	62.59	(58.69, 66.50)	86.97	99.7
Jiwani et al. (Nairobi), 2025	22	62.79	(58.82, 66.76)	89.97	99.7
Tassie et al., 2025	22	62.80	(58.83, 66.77)	89.88	99.7

PCMC = person-centered maternity care, CI = confidence interval.

### Subgroup analyses

Subgroup or moderator analyses were conducted by study country, study setting, publication year, sampling technique, measurement scale, and sample size to explore the sources of substantial heterogeneity. The pooled mean PCMC score showed numerical variation across the moderators.

Subgroup analysis by study country revealed a high pooled mean PCMC score in Kenya compared to Ethiopia. Under the common‑effect model, Ethiopian studies had a pooled mean PCMC score of 55.96, while Kenyan studies reported 65.82, with a statistically significant difference between groups (Q = 2176.1, *p* < 0.001). In the random‑effects model, Ethiopian studies reported a pooled mean PCMC score of 60.84 and Kenyan studies revealed 65.53; however, the difference was not statistically significant (Q = 2.11, p = 0.15). Heterogeneity remained very high in both subgroups (Ethiopia *I²* = 99.8%; Kenya *I²* = 97.9%) (Fig 9).

**Fig 9:**
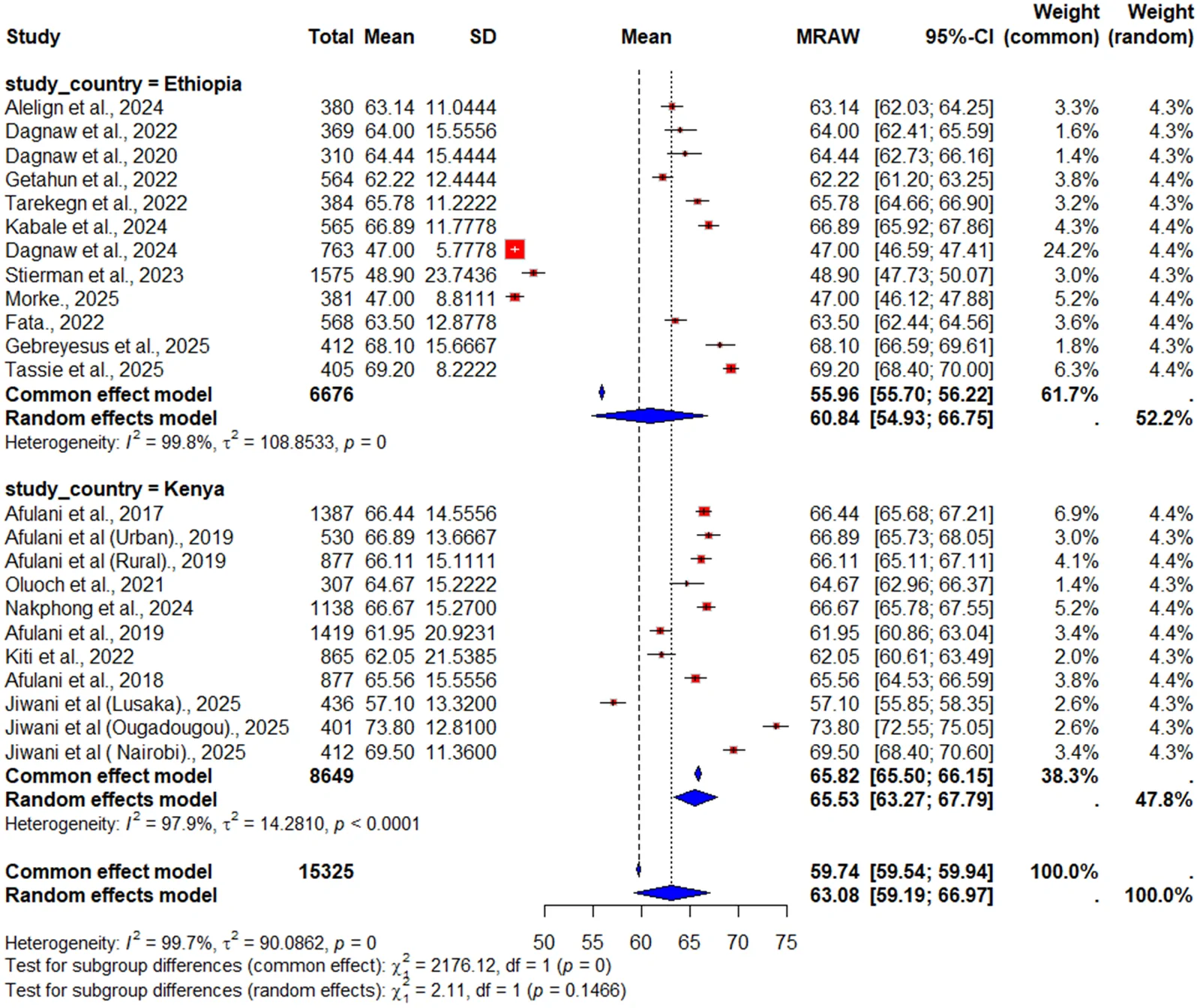
Subgroup forest plot of pooled mean person-centered maternity care score in East Africa by study country.

Subgroup analysis by sampling technique yielded a statistically significant between-subgroup difference under both common and random-effects model. In the common‑effect model, consecutive sampling studies had the highest pooled mean PCMC score (66.4), followed by multistage sampling (63.8), and systematic random sampling (56.4). Differences between groups were statistically significant (Q = 1423.3, p < 0.001). Under the random‑effects model, consecutive sampling remained highest (66.4), multistage sampling yielded 63.4 and systematic random sampling 62.2. Subgroup differences were also statistically significant (Q = 45.2, p < 0.001) (Fig 10).

**Fig 10:**
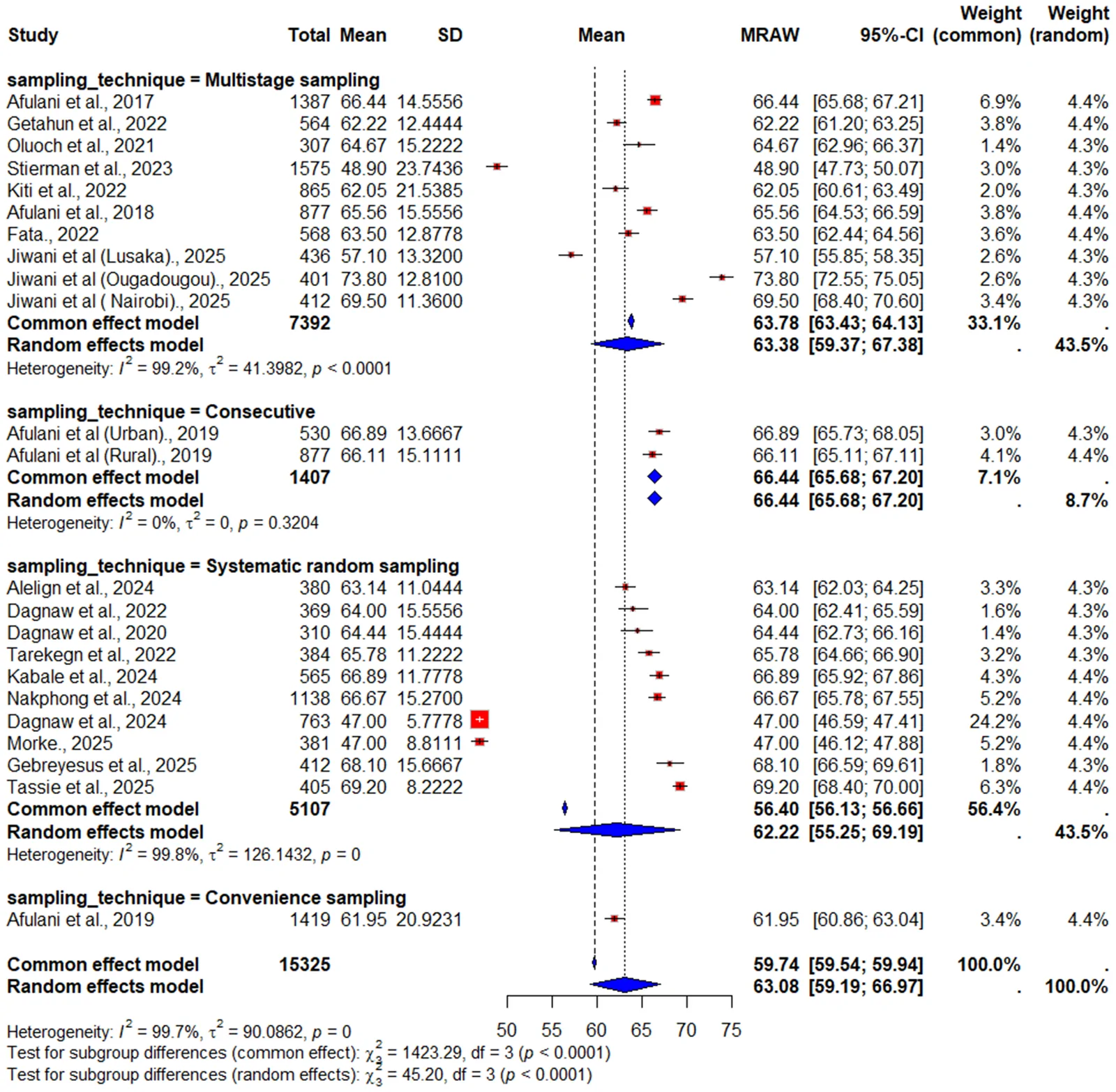
Subgroup forest plot of pooled mean person-centered maternity care score in East Africa by sampling technique.

Subgroup analysis by study setting revealed that under the common‑effect model, community‑based studies had a pooled mean PCMC score of **56.5,** institution‑based studies **59.56,** and mixed setting studies (institution and community-based) **64.2**, with a statistically significant difference between groups (Q = 201.1, p < 0.001). In the random‑effects model, community‑based studies yielded a mean PCMC score of **59.32**, institution‑based **63.59**, and mixed **64.03**, but subgroup differences were not statistically significant (Q = 0.59, p = 0.74). Heterogeneity remained very high across all settings (I^2^ > 96%) (Fig 11).

**Fig 11:**
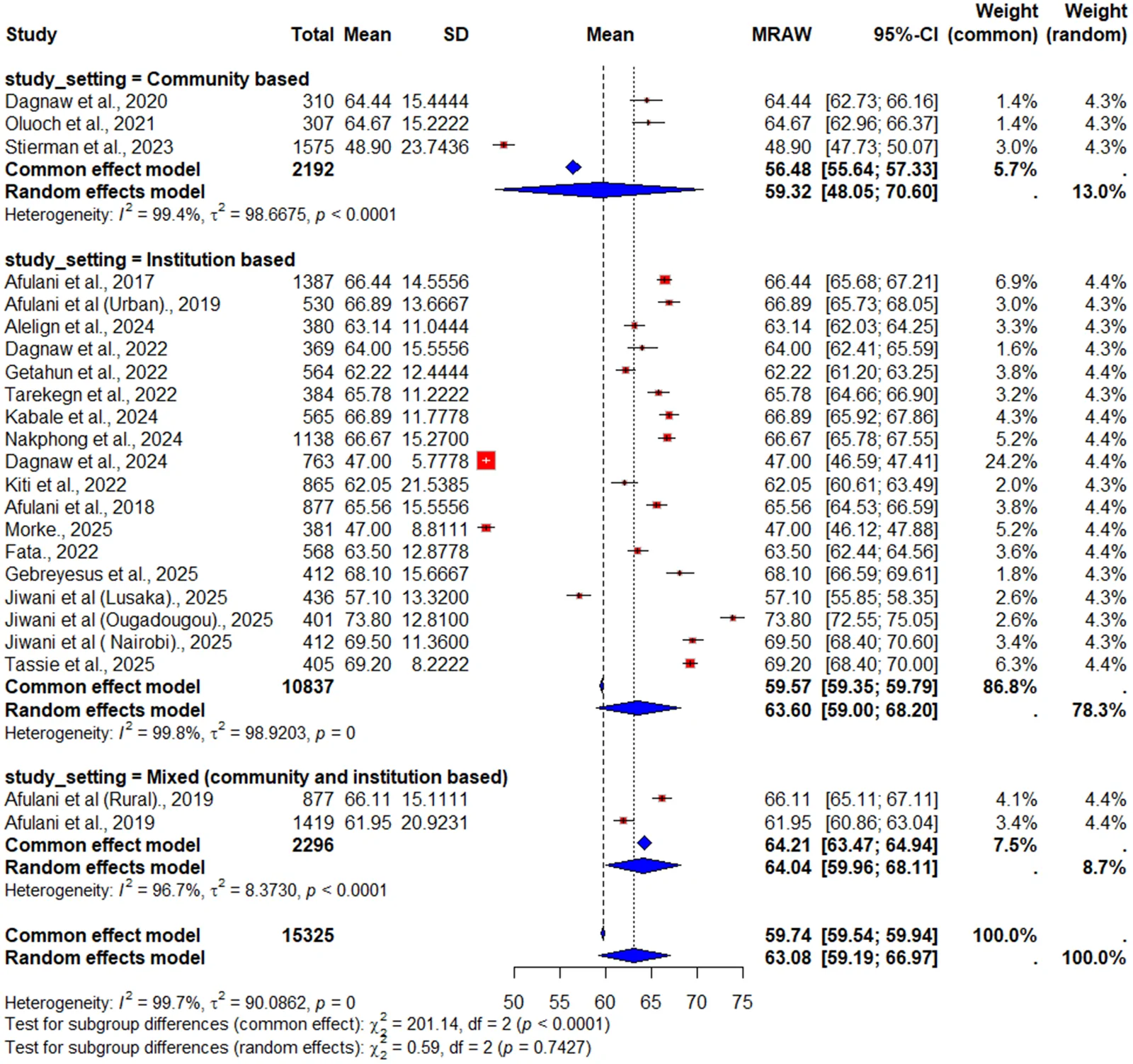
Subgroup forest plot of pooled mean person-centered maternity care in East Africa by study setting.

Subgroup analysis by publication year indicated that studies published before 2020 had a high pooled mean PCMC score (**65.6)**, while those published in 2020 or later reported **58.2** under the common‑effect model, with a statistically significant difference between groups (Q = 863.8, p < 0.001). In the random‑effects model, pre‑2020 studies yielded **65.4** and post‑2020 studies **62.4**, but subgroup differences were not statistically significant (Q = 1.34, p = 0.25). Heterogeneity remained very high in both categories (I^2^ > 92%) (Fig 12).

**Fig 12:**
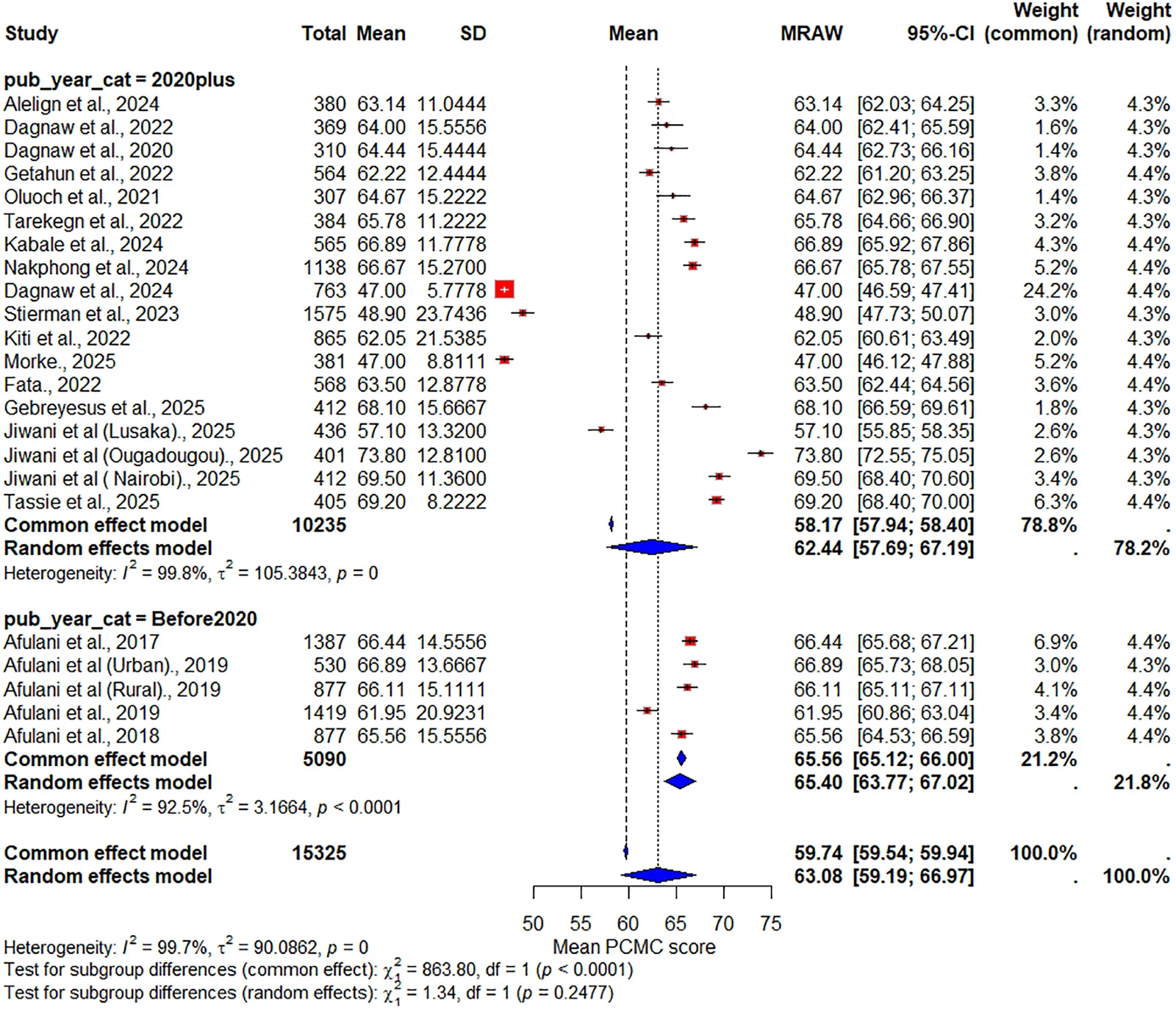
Subgroup forest plot of pooled mean person-centered maternity care in East Africa by publication year.

Subgroup analysis by sample size under the common‑effect model revealed that large sample studies had a low pooled mean PCMC score (57.5), while small sample studies reported a higher pooled mean score (63.8). The difference between groups was statistically significant (Q = 847.0, p < 0.001). In the random‑effects model, large‑sample studies yielded a pooled mean PCMC score of 61.6, compared to 64.5 for small‑sample studies. However, subgroup differences were not statistically significant (Q = 0.57, p = 0.45) (Fig 13).

**Fig 13:**
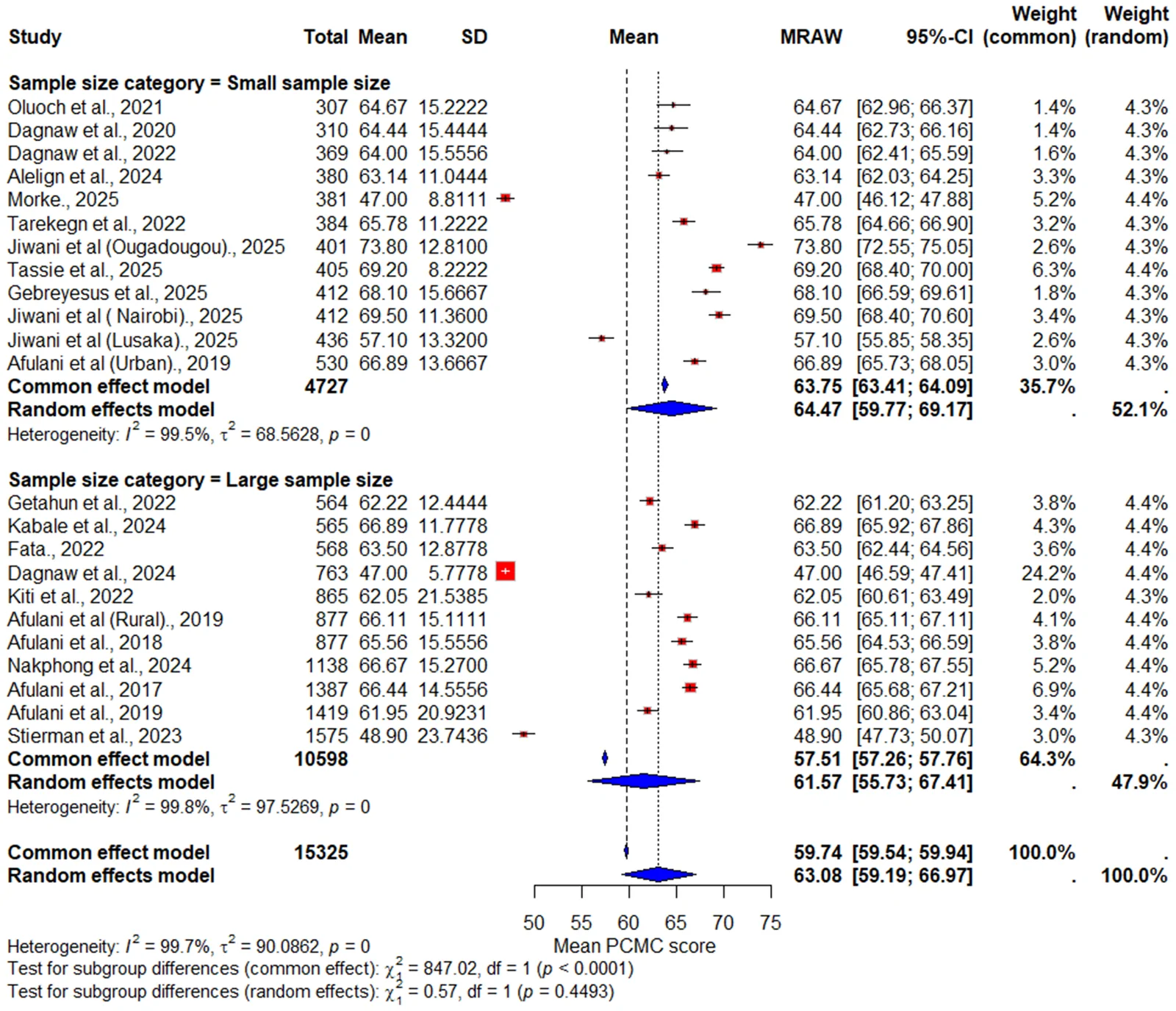
Subgroup forest plot of pooled mean person-centered maternity care in East Africa by sample size.

Subgroup analysis by measurement scale revealed studies using the 13‑item PCMC scale had a pooled mean score of **57.36** under the common‑effect model and **57.63** under the random‑effects model, while those using the 30‑item scale reported higher pooled means **(59.96** and **63.90**, respectively). The difference between scales was statistically significant under the common‑effect model (Q = 48.86, p < 0.001) but not under the random‑effects model (Q = 1.58, p = 0.21). Heterogeneity remained extreme in both subgroups (I^2^ > 99%) (Fig 14).

**Fig 14:**
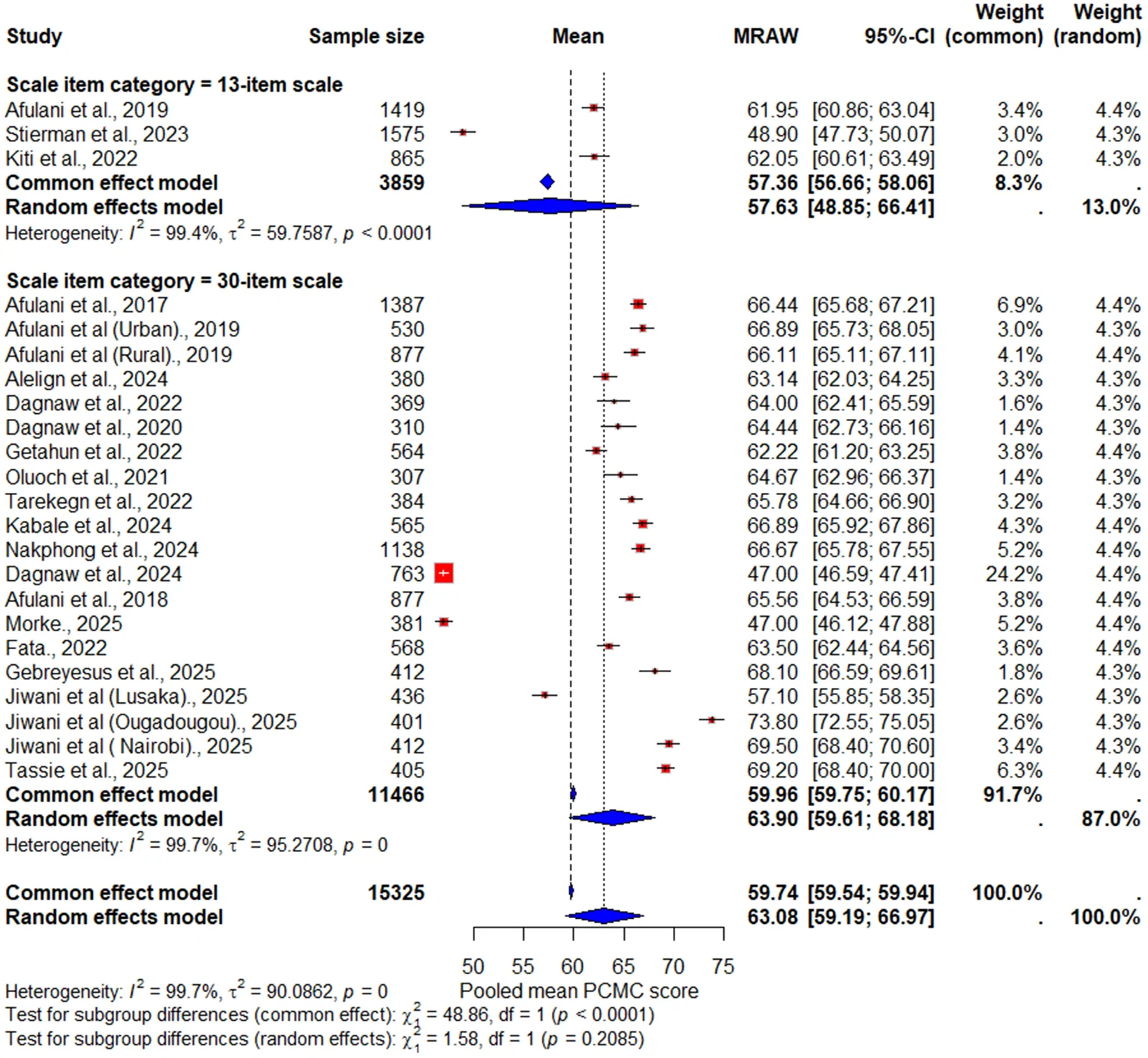
Subgroup forest plot of pooled mean person-centered maternity care in East Africa by measurement scale.

### Meta regression

Meta‑regression models to explore potential sources of heterogeneity examining study country, study setting, sampling technique, publication year, and sample size showed that none of these covariates were statistically significant moderators of PCMC (all *p* > 0.05). Study country explained the largest proportion of variance (R^2^ = 21.9%), with Kenyan studies reporting slightly higher scores than Ethiopian studies, but this difference was not significant. Study setting, sampling technique, publication year, and sample size each accounted for little to no heterogeneity (R^2^ range 0–4.8%). Across all models, residual heterogeneity remained extreme (I^2^ > 99%), indicating that these study characteristics do not meaningfully explain variability in pooled PCMC scores.

### Predictors of PCMC in East Africa

This systematic review and meta-analysis showed that nighttime delivery, late ANC initiation, and rural residence were associated with poorer PCMC. While attaining secondary as well as college and above education were significantly associated with better PCMC during childbirth (Fig 15).

**Fig 15:**
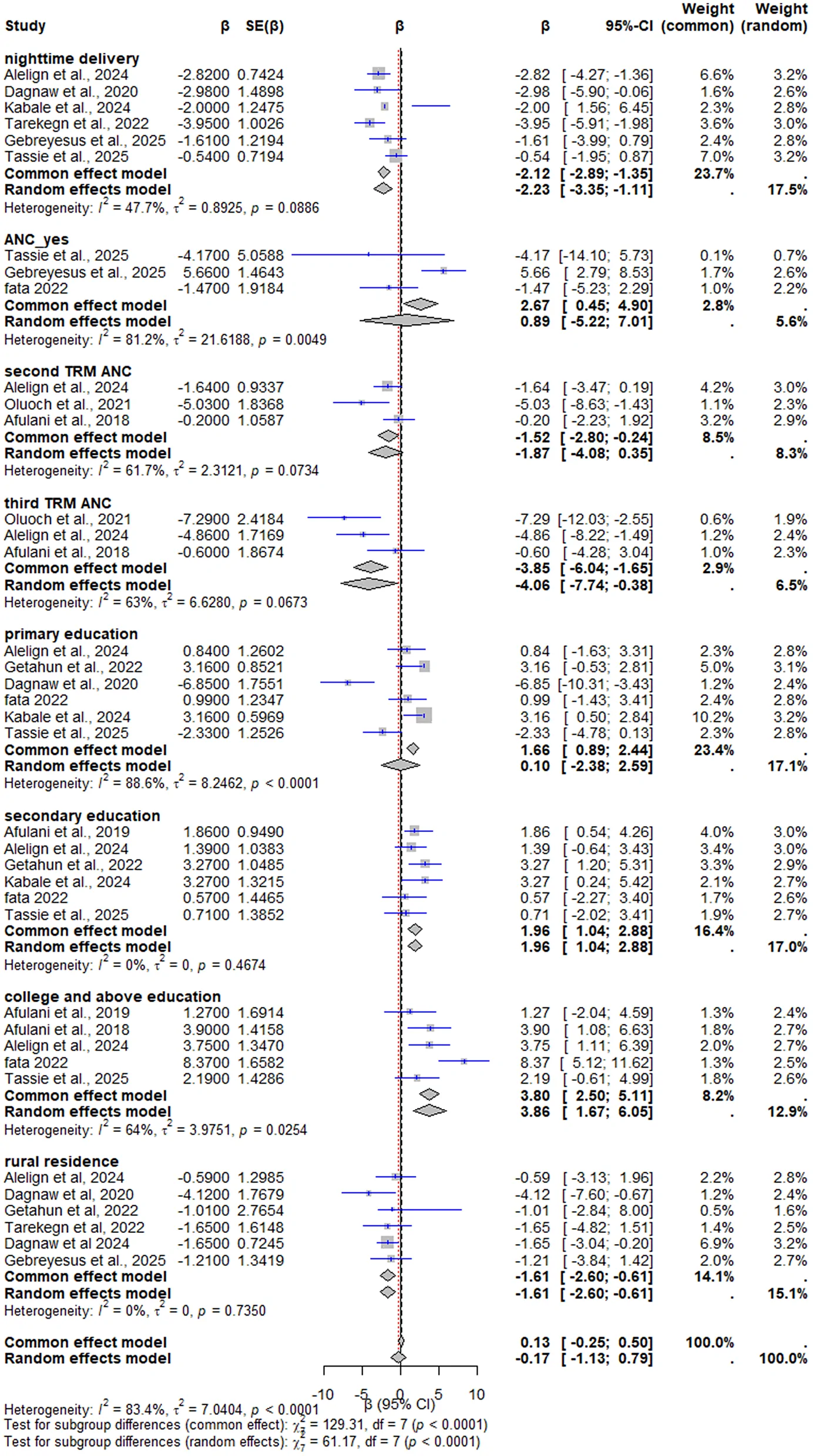
Forest plot for pooled predictors of person-centered maternity care during childbirth in East Africa.

Forest plot showing pooled β coefficients for predictors of person-centered maternity care in East Africa. Squares represent study-specific effect estimate for each predictor, with square size proportional to invers-variance weight and horizontal line indicate 95% CIs. Diamonds represent the pooled effect estimates for each predictor derived from random-effects meta-analysis model, with diamond width represent the 95% CI. The vertical reference line at β = 0 denotes no association or the null value. Predictors with CIs not crossing the vertical reference line are statistically significantly associated with person-centered maternity care.

The pooled beta coefficient (β) from six studies [10,15,17,18,37,38] indicates that women who gave birth during nighttime experienced a 2.23-unit decrease in PCMC score as compared to those who gave birth during daytime (pooled β = -2.23; 95% CI: -3.35, -1.11). The pooled β of three studies [4,10,41] indicated that women who commenced ANC during third trimester received a 4.06-unit lower PCMC compared to those who started early (pooled β = -4.06, 95% CI: -7.74, -0.38). The pooled β from six studies [10,16–18,35,38] revealed that women residing in rural area experienced a 1.61-unit lower PCMC compared to their urban counterparts (pooled β = -1.61; 95% CI: -2.60, -0.61).

The pooled β from six studies [10,15,16,18,25,37] suggests that women with a secondary education received 1.96 units higher PCMC compared to women with no formal education (pooled β = 1.96, 95% CI: 1.04, 2.88). In addition, the pooled β from five studies [4,10,25,37,40] revealed that women with college and above education received a 3.86-unit higher PCMC compared to their counterparts (pooled β = 3.86, 95% CI: 1.67, 6.05).

## Discussion

This systematic review and meta-analysis was aimed to provide a comprehensive assessment of the pooled mean PCMC score and its domains (dignity and respect, communication and autonomy, and supportive care) as well as its predictors among women who gave birth in East Africa.

This review found a moderate pooled mean PCMC score in East Africa (63.08%); however, the estimate should be interpreted cautiously because heterogeneity was extremely high. The wide variation across studies indicates that women’s childbirth experiences differ considerably across settings. The pooled score is higher than that observed in a previous meta-analysis conducted in Ethiopia (57.92%) [44]. This variation may be partly attributed by inclusion of studies from both Ethiopia and Kenya, depicting cross-country difference in maternity care as well as policy implementation. Additionally, the variation may be explained by the larger number of studies and sample size in the current study, which may reveal a more representative pooled estimate. Moreover, our review incorporates more recent studies conducted after 2025, allowing it to capture the latest evidence, trends, and advancement in maternity care, reflecting the most up-to-date and comprehensive understanding in the field.

However, the finding of this review is in line with an interventional study conducted in Kenya, where the post intervention PCMC score was 63.98% [33]. This might be due to similarity in measurement tool and study population. While this finding suggests a moderate level of adherence to practices of PCMC during childbirth, it falls short of an interventional study in India [45] and that of WHO benchmarks [9]. This shortfall suggests that efforts to enhance PCMC are still needed across East African health facilities to meet the standards set by WHO. Thus, targeted interventions such as healthcare provider training, appropriate resource allocation and monitoring of care quality are recommended to enhance the quality of maternity care in East Africa.

The pooled estimate for dignity and respect in this study was 78.05% (95% CI: 74.43, 81.67), which indicates that health facilities in East Africa succeed in delivering care that is respectful and dignified. This is consistent with an interventional study conducted in Kenya [33] and shows some alignment with a similar study in India [45]. This finding also aligns somewhat with the WHO standard 4, which considers respectful care—including being treated with dignity, not experiencing verbal or physical abuse, and having privacy—essential for a positive childbirth experience [9]. However, although this result is promising, it still indicates that nearly 20% of women might experience care that does not fully meet this domain, a gap that is significant given WHO’s goal of universal respectful treatment.

The pooled mean score of communication and autonomy was notably lower, at 55.29% (95% CI: 48.66, 61.92), which is in agreement with an interventional study done in Kenya [33] but far lower than an interventional study in India [45] and WHO standard 5 [9]. The low score in this domain might reflect provider–patient power imbalances, heavy workloads, inadequate communication skills, insufficient training, and limited opportunities for women to participate in decisions regarding their care. The consistently low score across studies could also suggest systems-level weakness rather than an isolated provider issue. Effective communication and the provision of autonomy are core principles of standard 5 of the WHO framework for quality maternity care, emphasizing that women should be actively involved in decisions during childbirth, informed about procedures, and encouraged to ask questions [9].

The pooled mean score of supportive care was 62.59% (95% CI: 59.44, 65.73), which is in line with an interventional study done in Kenya, where the post intervention score of supportive care was 64.01% [33] but far lower than a similar study in India [45] and standard 6 of the WHO quality care framework [9]. Supportive care, as highlighted by standard 6 of the WHO quality care framework, includes physical comfort, emotional support, and practical assistance during childbirth [9]. It is crucial not only for the well-being of the mother but also for the health and safety of both mother and newborn. The fact that approximately 40% of women in East African health facilities did not receive adequate supportive care is concerning. This shortfall suggests that healthcare providers may not always be equipped or adequately trained to offer consistent supportive care, underscoring the need for interventions that equip healthcare workers with the skills and resources to provide this level of care effectively.

Measurement variability between the 13‑item and 30‑item PCMC scales likely contributed to heterogeneity in pooled estimates. While both instruments assess the same overarching construct, the shorter scale condenses domains and may omit nuances captured by the full version. In addition, cultural and system‑level differences across study settings—such as differences in health system, provider–patient communication, and sociocultural expectations—further influence PCMC scores.

Women who delivered at night had, on average, a 2.23-point lower PCMC score compared to those who delivered during daytime (β = -2.23; 95% CI: -3.35, -1.11). This finding is supported by previous studies conducted in Ethiopia [44] and other LMIC [10,15,17,18]. This pattern likely reflects a form of temporal inequity in maternity care quality. Possible explanations could be reduced staffing, provider fatigue, high workload, weak supervision, and resource constraints during night shifts, which may reduce the focus on PCMC; similar time-of-day variations in healthcare outcomes and service utilization patterns have been reported in other public health contexts [46].

In accordance with previous evidence [47], this study highlights that women who started ANC during third trimester of pregnancy received 4.06 unit lower PCMC scores compared to those who commenced in the first trimester (pooled β = -4.06, 95% CI: -7.74, -0.38). This could be due to the fact that late initiation of ANC is associated with unfamiliarity of the healthcare system likely impede to get the support from health care providers.

This finding revealed that women who attain secondary education received, on average, a 1.96-point higher PCMC score compared to those who have no formal education (pooled β = 1.96, 95% CI: 1.04, 2.88). Additionally, college and above educated women received a 3.86-unit higher PCMC compared to their counterparts (pooled β = 3.86, 95% CI: 1.67, 6.05). This finding is consistent with studies done in other LMIC [25,41]. This could be due to the fact that educated women are more likely to have a good health-seeking behavior [48] and effectively communicate with care providers to ensure a better experience of care [41].

This finding indicated that rural resident women received, on average, a 1.61-point lower PCMC score compared to urban dwellers (pooled β = -1.61, 95% CI: -2.60, -0.61). This finding is consistent with previous studies [17,35]. This disparity may be attributed to the fact that rural resident women often have lower socioeconomic status or face cultural norms that limit their ability to advocate for themselves in healthcare settings. Such factors can negatively influence their experiences and expectations of PCMC compared to women in urban settings who may feel more empowered to request high-quality, respectful care [49].

### Strengths and limitations

Strengths of this review include the comprehensive search strategy employed, inclusion of both published and unpublished studies, adherence to PRISMA and MOOSE guidelines, and separate synthesis of PCMC domains and predictors

However, several limitations should be acknowledged. First, heterogeneity was high, limiting the interpretability of pooled estimates. Second, evidence of publication bias was detected, and trim-and-fill analysis suggested that the pooled estimate may be overestimated. Third, only studies from Ethiopia and Kenya were identified; therefore, the findings may not be generalizable to all East African countries. Fourth, all included studies were cross-sectional, precluding causal inference. Finally, while scores were harmonized to a common 0–100 metric, differences between the 13‑item short form and the 30‑item comprehensive PCMC scale may have introduced measurement variability. Although both instruments assess the same overarching construct, their differing item coverage and granularity likely contributed to residual heterogeneity in pooled estimates.

## Conclusion and recommendations

PCMC during childbirth in East Africa remains suboptimal, particularly in the domains of communication, autonomy, and supportive care, compared to WHO benchmarks, Nighttime delivery, delayed ANC initiation, and rural residence were associated with lower PCMC scores, whereas women’s education was associated with higher scores. However, these findings should be interpreted with caution because of substantial heterogeneity, publication bias, and limited geographic representation.

Future initiatives should focus on strengthening provider training on communication skills and shared decision-making, simulation-based training, stronger night-shift staffing and supervision, and integrating PCMC indicators within national maternal health quality monitoring systems. Implementing task-shifting models to expand community-level delivery of ANC counseling, birth preparedness education, and supportive care to address rural disparities and delayed ANC initiation is essential. In addition, community engagement strategies such as participatory women’s groups, health education, and community–facility linkage mechanisms, can improve awareness, trust, and utilization maternity services. Future research should focus on implementation science and intervention studies, aimed at improving communication and autonomy domain.

## Supporting information

S1 Checklist PRISMA 2020 checklist. (DOCX)

S1 Table Full search strategies for databases. (DOCX)

S2 Table Studies identified in the literature search. (DOCX)

S1 Data Full extracted datasets. (XLSX)

S3 Table Risk-of-bias judgments and domain level assessment using JBI critical appraisal checklist. (DOCX)

## Abbreviations

ANC: Antenatal Care
CI: Confidence interval
DOAJ: Directory of Open Access Journals
JBI: Joanna Briggs Institute
LMICs: Low- and Middle-Income Countries
PCMC: Person-Centered Maternity Care
PRISMA: Preferred Reporting Items for Systematic Reviews and Meta-Analysis
SD: Standard deviation
WHO: World Health Organization
MOOSE: Meta-Analysis of Observational Studies in Epidemiology
