# Supplementary material for: Prevalence of person-centered maternity care during childbirth and its predictors among women who gave birth in health facilities of East Africa: A systematic review and meta-analysis
Source: PLOS Glob Public Health. 2026 Sep 29;6(9):e0005909. doi: 10.1371/journal.pgph.0005909 (PMC13623155; doi:10.1371/journal.pgph.0005909)
Supplement: Supplementary file 2 [file pgph.0005909.s002.docx]

| **Databases** | **Searching terms** | **Refined by** | **Number of studies** | **Last searching date** |
| --- | --- | --- | --- | --- |
| PubMed | ((((((((((((((((Person-centered care[Title/Abstract]) OR (Person-centered maternity care[Title/Abstract])) OR (Patient-centered care[Title/Abstract])) OR (Patient-centered care[MeSH Terms])) OR (Woman centered care[Title/Abstract])) OR (Client centered care[Title/Abstract])) OR (Childbirth experience[Title/Abstract])) OR (Maternity care[Title/Abstract])) OR (Childbirth[Title/Abstract])) OR (Quality of care[Title/Abstract])) OR (Communication[Text Word])) OR (Autonomy[Text Word])) OR (Dignity[Text Word])) OR (Respect[Text Word])) OR (Supportive care[Text Word])) AND (Africa, Eastern[MeSH Terms])) NOT (Systematic review[Title/Abstract]) | **Female, Humans, publication date from** 2017/01/01 **–** 2025/12/24 | 2,596 | 2025/12/24 |
| Research4Life | (Person-centered maternity care) AND (Burundi OR Comoros OR Djibouti OR Ethiopia OR Eritrea OR Kenya OR Seychelles OR Rwanda OR Somalia OR South Sudan OR Sudan OR Tanzania OR Uganda) | Publication date: 01/01/2017-12/24/2025 | 95 |  |
| ScienceDirect | Person-centered maternity care AND Eastern Africa | Publication date: 2017-2025/12/24 | 436 |  |
| Google scholar | With all of the words: Person centered care; With exact phrase: "Person-centered maternity care"; With at least one of the words: Burundi Comoros Djibouti Ethiopia Eritrea Kenya Seychelles Rwanda Somalia South Sudan Sudan Tanzania Uganda;  Where my words occur: anywhere in the article. | Publication date: 2017-2025 | 582 |  |
| Directory Of Open Access Journals (DOAJ) | (Person-centered maternity care) AND (Burundi OR Comoros OR Djibouti OR Ethiopia OR Eritrea OR Kenya OR Seychelles OR Rwanda OR Somalia OR South Sudan OR Sudan OR Tanzania OR Uganda) | Year of publication: 2017 - 2025 | 80 |  |
| Total retrieved articles | | | 3,789 |  |
| Studies eligible for the final systematic review and meta-analysis | | | 23 |  |

S1 Table. Full search strategies for databases.
