# Supplementary material for: Prevalence of person-centered maternity care during childbirth and its predictors among women who gave birth in health facilities of East Africa: A systematic review and meta-analysis
Source: PLOS Glob Public Health. 2026 Sep 29;6(9):e0005909. doi: 10.1371/journal.pgph.0005909 (PMC13623155; doi:10.1371/journal.pgph.0005909)
Supplement: Supplementary file 3 [file pgph.0005909.s003.docx]

S2 Table. Studies identified in the literature search and screened for eligibility at full-text review (n = 30)

| Study No. | Author, year | Study title | Country | Status (included /  excluded) | Reason for exclusion | Access information (if unpublished) |
| --- | --- | --- | --- | --- | --- | --- |
| 1 | Afulani et al., 2017 | Development of a tool to measure person-centered maternity care in developing settings: validation in a rural and urban Kenyan population | Kenya | Included | - | - |
| 2 | Afulani et al., (Urban) 2019 | Person-centred maternity care in low-income and middle-income countries: analysis of data from Kenya, Ghana, and India | Kenya | Included | - | - |
| 3 | Afulani et al., (Rural) 2019) | Person-centred maternity care in low-income and middle-income countries: analysis of data from Kenya, Ghana, and India | Kenya | Included | - | - |
| 4 | Alelign et al., 2024 | Person-centered maternity care during childbirth and associated factors among mothers who gave birth at public health institutions of Debre Markos town, Northwest Ethiopia, 2022: A cross-sectional study | Ethiopia | Included | - | - |
| 5 | Dagnaw et al., 2022 | Person-centered Maternity Care Among Mothers Who Gave Birth in South Wollo Zone Public Hospitals, Northeastern Ethiopia: A Mixed-method Study | Ethiopia | Included | - | - |
| 6 | Dagnaw et al., 2020 | Determinants of person-centered maternity care at the selected health facilities of Dessie town, Northeastern, Ethiopia: community-based cross-sectional study | Ethiopia | Included | - | - |
| 7 | Getahun et al., 2022 | Person-centered care during childbirth and associated factors among mothers who gave birth at health facilities in Hawassa city administration Sidama Region, Southern Ethiopia | Ethiopia | Included | - | - |
| 8 | Oluoch et al., 2021 | Examining person-centered maternity care in a peri-urban setting in Embakasi, Nairobi, Kenya | Kenya | Included | - | - |
| 9 | Tarekegne, Giru and Mekonnen., 2022 | Person-centered maternity care during childbirth and associated factors at selected public hospitals in Addis Ababa, Ethiopia, 2021: a cross-sectional study | Ethiopia | Included | - | - |
| 10 | Kabale et al., 2024 | Person-centered maternity care during childbirth and associated factors at public hospitals in central Ethiopia | Ethiopia | Included | - | - |
| 11 | Nakphong et al., 2024 | Integrating support persons into maternity care and associations with quality of care: a postpartum survey of mothers and support persons in Kenya | Kenya | Included | - | - |
| 12 | Dagnaw, Bizuneh and Kassie., 2024 | Person-centered maternity care and associated factors among women who give birth at public hospitals in South Gondar zone, North West Ethiopia, 2023 | Ethiopia | Included | - | - |
| 13 | Afulani et al., 2019 | Toward the development of a short multi‐country person‐centered maternity care scale | Kenya | Included | - | - |
| 14 | Stierman et al., 2023 | Understanding variation in person-centered maternity care: results from a household survey of postpartum women in 6 regions of Ethiopia | Ethiopia | Included | - | - |
| 15 | Kiti et al., 2022 | Continuous labor support and person-centered maternity care: a cross-sectional study with women in rural Kenya | Kenya | Included | - | - |
| 16 | Afulani et al., 2018 | Predictors of person-centered maternity care: the role of socioeconomic status, empowerment, and facility type | Kenya | Included | - | - |
| 17 | Morke., 2025 | Prevalence and Associated Factors of Person- Centred Maternity Care Among Women Who Gave Birth in Public Hospitals of West Arsi Zone , Oromia , Ethiopia, 2023 | Ethiopia | Included | - | <https://assets-eu.researchsquare.com/files/rs-6535872/v1/61079f6d-b2c0-4427-b07f-e4506b4cc8c8.pdf?c=1748882491> |
| 18 | Fata., 2022 | Person-Centered Maternity Care During Childbirth And Associated Factors Among Mothers Who Gave Birth At Arsi Zone Hospitals, Oromia, Ethiopia | Ethiopia | Included | - | <http://irlib.ambou.edu.et/xmlui/> |
| 19 | Gebreyesus et al., 2025 | Person-centered maternity care and its associated factors during childbirth at selected public hospitals in Eastern Ethiopia: a cross-sectional study | Ethiopia | Included | - | - |
| 20 | Jiwani et al (Lusaka)., 2025 | Levels and determinants of person-centred maternity care among women living in urban informal settlements: evidence from client exit surveys in Nairobi, Lusaka and Ouagadougou | Kenya | Included | - | <https://www.medrxiv.org/content/10.1101/2025.03.04.25323334v1> |
| 21 | Jiwani et al (Ougadougou)., 2025 | Levels and determinants of person-centred maternity care among women living in urban informal settlements: evidence from client exit surveys in Nairobi, Lusaka and Ouagadougou | Kenya | Included | - | <https://www.medrxiv.org/content/10.1101/2025.03.04.25323334v1> |
| 22 | Jiwani et al (Nairobi)., 2025 | Levels and determinants of person-centred maternity care among women living in urban informal settlements: evidence from client exit surveys in Nairobi, Lusaka and Ouagadougou | Kenya | Included | - | <https://www.medrxiv.org/content/10.1101/2025.03.04.25323334v1> |
| 23 | Tassie et al., 2025 | Determinants of person-centered maternity care during child birth at the selected hospitals of South Gondar Zone, Northwest Ethiopia: mixed cross-sectional study | Ethiopia | Included | - | - |
| 24 | Afulanu et al., 2023 | Person‐centred sexual and reproductive health: A call for standardized measurement | Kenya | Excluded | Outcome of interest not reported | - |
| 25 | Stierman et al., 2022 | Evaluating childbirth service readiness and the provision of person-centered maternity care at health facilities in Ethiopia | Ethiopia | Excluded | Full text article inaccessible | - |
| 26 | Sudhinaraset et al., 2019 | Providers and women's perspectives on person-centered maternity care: a mixed methods study in Kenya | Kenya | Excluded | Outcome of interest not reported  No statistical analysis (only percentage distribution reported, no effect size measure | - |
| 27 | Sudhinaraset et al., 2020 | Association between person-centered maternity care and newborn complications in Kenya | Kenya | Excluded | Outcome of interest not reported | - |
| 28 | Sudhinaraset et al., 2021 | Person-centered maternity care and postnatal health: associations with maternal and newborn health outcomes | Kenya | Excluded | Outcome of interest was not reported and different effect size measures (RR) | - |
| 29 | Sudhinaraset et al., 2023 | Can a quality improvement intervention improve person-centred maternity care in Kenya? | Kenya | Excluded | Different study design ( RCT) and different effect size measure | - |
| 30 | Jiwani et al., 2025 | Hidden Cities, Hidden Gaps: Measuring Facility Readiness for Maternal and Newborn Health Services and its Association with Person-Centered Maternity Care in Urban Informal Settlements of Nairobi, Lusaka and Ouagadougou cities. | Kenya | Excluded | different outcome of interest | - |
