# Supplementary material for: Prevalence of person-centered maternity care during childbirth and its predictors among women who gave birth in health facilities of East Africa: A systematic review and meta-analysis
Source: PLOS Glob Public Health. 2026 Sep 29;6(9):e0005909. doi: 10.1371/journal.pgph.0005909 (PMC13623155; doi:10.1371/journal.pgph.0005909)
Supplement: Supplementary file 4 [file pgph.0005909.s005.docx]

S3 Table. Risk-of-bias judgments and domain-level assessments of included studies using the Joanna Briggs Institute (JBI) Critical Appraisal Checklist for studies reporting prevalence data.

| **No** | **Study** | **Study country** | **JBI critical appraisal checklist for prevalence studies** | | | | | | | | | | |
| --- | --- | --- | --- | --- | --- | --- | --- | --- | --- | --- | --- | --- | --- |
|  |  |  | **Q1** | **Q2** | **Q3** | **Q4** | **Q5** | **Q6** | **Q7** | **Q8** | **Q9** | **Total score** | **Overall risk of bias** |
| 1 | Afulani et al., 2017 | Kenya | Y | Y | Y | Y | Y | Y | Y | Y | Y | 9/9 | Low |
| 2 | Afulani et al., 2019 | Kenya | Y | Y | Y | Y | N | Y | Y | Y | Y | 8/9 | Low |
| 3 | Afulani et al., (urban) 2019 | Kenya | Y | Y | Y | Y | N | Y | Y | Y | Y | 8/9 | Low |
| 4 | Afulani et al., (rural) 2019 | Kenya | Y | Y | Y | Y | N | Y | Y | Y | Y | 8/9 | Low |
| 5 | Oluoch-Aridi et al., 2021 | Kenya | Y | N | Y | Y | N | Y | Y | Y | Y | 7/9 | Low |
| 6 | Jiwani et al (Lusaka)., 2025 | Kenya | Y | Y | Y | Y | Y | Y | Y | Y | Y | 9/9 | Low |
| 7 | Jiwani et al (Ougadougou)., 2025 | Kenya | Y | Y | Y | Y | Y | Y | Y | Y | Y | 9/9 | Low |
| 8 | Jiwani et al (Nairobi)., 2025 | Kenya | Y | Y | Y | Y | Y | Y | Y | Y | Y | 9/9 | Low |
| 9 | Alelign et al., 2024 | Ethiopia | Y | Y | Y | Y | Y | Y | Y | Y | Y | 9/9 | Low |
| 10 | Nakphong et al., 2024 | Kenya | Y | Y | Y | Y | Y | Y | Y | Y | Y | 9/9 | Low |
| 11 | Kiti et al., 2022 | Kenya | Y | Y | Y | Y | Y | Y | Y | Y | Y | 9/9 | Low |
| 12 | Afulani et al., 2018 | Kenya | Y | Y | Y | Y | Y | Y | Y | Y | Y | 9/9 | Low |
| 13 | Dagnaw, Bizuneh and Kassie., 2024 | Ethiopia | Y | Y | Y | Y | N | Y | Y | Y | Y | 8/9 | Low |
| 14 | Dagnaw et al., 2022 | Ethiopia | Y | Y | Y | Y | Y | Y | Y | Y | Y | 9/9 | Low |
| 15 | Dagnaw et al., 2020 | Ethiopia | N | Y | Y | Y | N | Y | Y | Y | Y | 7/9 | Low |
| 16 | Getahun et al., 2022 | Ethiopia | Y | Y | Y | Y | Y | Y | Y | Y | Y | 9/9 | Low |
| 17 | Tarekegne et al., 2022 | Ethiopia | Y | Y | Y | Y | Y | Y | Y | Y | Y | 9/9 | Low |
| 18 | Stierrman et al., 2023 | Ethiopia | Y | Y | Y | Y | Y | Y | Y | Y | Y | 9/9 | Low |
| 19 | Tassie et al., 2025 | Ethiopia | Y | Y | Y | Y | Y | Y | Y | Y | Y | 9/9 | Low |
| 20 | Kabale et al., 2024 | Ethiopia | Y | Y | Y | Y | Y | Y | Y | Y | Y | 9/9 | Low |
| 21 | Gebreyesus et al., 2025 | Ethiopia | Y | Y | Y | Y | Y | Y | Y | Y | Y | 9/9 | Low |
| 22 | Fata., 2022 | Ethiopia | Y | Y | Y | Y | Y | Y | Y | Y | Y | 9/9 | Low |
| 23 | Morke et al., 2025 | Ethiopia | Y | Y | Y | Y | Y | Y | Y | Y | Y | 9/9 | Low |

Note:

(Y - Yes) =1, and (N - No, U - Unclear, NA-Not applicable) = 0

Q1 = Was the sample frame appropriate to address the target population?

Q2 = Were study participants sampled in an appropriate way?

Q3 = Was the sample size adequate?

Q4 = Were the study subjects and the setting described in detail?

Q5 = Was the data analysis conducted with sufficient coverage of the identified sample?

Q6 = Were valid methods used for the identification of the condition?

Q7 = Was the condition measured in a standard, reliable way for all participants?

Q8 = Was there appropriate statistical analysis?

Q9 = Was the response rate adequate, and if not, was the low response rate managed appropriately?
