# Supplementary material for: Prevalence of person-centered maternity care during childbirth and its predictors among women who gave birth in health facilities of East Africa: A systematic review and meta-analysis
Source: PLOS Glob Public Health. 2026 Sep 29;6(9):e0005909. doi: 10.1371/journal.pgph.0005909 (PMC13623155; doi:10.1371/journal.pgph.0005909)
Supplement: Supplementary file 5 [file pgph.0005909.s007.docx]

# **Response to Reviewers**

We sincerely thank you Dear Editor and Reviewers for the careful evaluation and constructive and insightful feedback. We have carefully revised our manuscript to address each concern. The revisions have now significantly improved the methodological transparency, interpretation, and reporting of findings.

Major revisions include:

- Reframing the outcome throughout the manuscript from “prevalence” to “pooled mean PCMC score” where appropriate.
- Expanding the Introduction with updated regional maternal mortality and quality-of-care data.
- Providing justification for restricting studies to 2017 onward.
- Justifying the JBI quality threshold and clarifying the quality appraisal process.
- Adding meta-regression analyses to explore sources of heterogeneity.
- Revising the interpretation of the very high heterogeneity and moderating conclusions.
- Adding sensitivity analyses and expanded discussion regarding the 13-item versus 30-item PCMC scales.
- Strengthening the discussion and recommendations, especially regarding communication/autonomy and nighttime delivery.
- Preparing supplementary files containing the full extracted dataset, excluded studies with reasons, quality appraisal tables, leave-one-out analyses, and detailed heterogeneity statistics.

Below are the point-by-point responses to the Editor and Reviewers. All changes are highlighted in the tracked-changes version of the manuscript.

# Response to Editor

**Comment #1: Interpretation of heterogeneity and validity of pooled estimates**

**Response:** Thank you for your insightful suggestions. We accept and substantially revised the Results, Discussion, and Limitations sections to address the implications of the extremely high heterogeneity. Specifically:

1. We now clearly state that the pooled mean PCMC score should be interpreted cautiously because heterogeneity remained very high across studies.
2. We expanded the interpretation of possible sources of heterogeneity, including:
   - Differences between study countries
   - Differences in study setting
   - Differences in sampling technique
   - Variability in PCMC measurement instruments
   - Differences in publication year, and
   - Differences in sample size
3. We further performed meta-regression analyses by study country, study setting, sampling technique, publication year, and sample size.
4. We added a leave-one-out sensitivity analyses table and explicitly note that no single study substantially changed the pooled estimate, suggesting that heterogeneity is likely due to genuine between-study differences rather than one influential study.
5. We revised the Discussion and Conclusion section to avoid overinterpretation

**Comment #2: Methodological transparency and rigor**

**2a. Justification for restricting included studies to those published from 2017 onward**

**Response:** Thank you for your valuable suggestions. Studies published from 2017 onward were included because the validated PCMC scale was first introduced and operationalized in 2017. Restricting the review to this period ensured comparability of the construct and avoided inclusion of studies that used non-standardized or conceptually different measures of maternity care experience. We also note that the earliest eligible PCMC studies in East Africa appeared after 2017.

**2b. Justification of the JBI quality assessment cutoff**

**Response:** We sincerely thank you for your insightful observation. We agree that the previous cutoff was insufficiently justified. We revised in the Methods section to explain that the JBI checklist contains nine items, with each item rated as “Yes”, “No”, “Unclear”, or Not applicable. While we report the score of individual items and total scores (out of 9) for transparency, JBI emphasizes that appraisal should be based on reviewer judgment rather than rigid threshold. Accordingly, for inclusion of studies we did not consider numerical cutoff; instead, we used the checklist to identify potential risk of bias (as low risk of bias, moderate risk of bias, and high risk of bias).

We have provided a detailed study-level risk-of-bias table supplementary information file, including domain-specific judgments for all nine items of the Joanna Briggs Institute critical appraisal checklist and an overall risk-of-bias assessment for each included study.

**2c. Consideration of additional analyses (meta-regression)**

**Response:** Thank you for your constructive feedback. We accept and added meta-regression analyses using the following covariates: study country, publication year, study setting, and sample size. However, we could not perform meta-regression by scale type due to the limited number of studies in the 13-item scale (only 3) as at least 10 studies are need per moderator.

The meta-regression results are now been added in the Methods section and reported in the Results section.

**2d. More explicit reporting and justification of statistical approaches used**

**Response:** We thank you for the constructive feedback. We expanded the Statistical Analysis subsection to provide a clearer rationale for:

- Use of the random-effects model
- Use of the DerSimonian–Laird estimator
- Use of rescaling to a common 0–100 scale
- Use of subgroup analysis and meta-regression
- Use of leave-one-out sensitivity analysis

**Comment #3: Measurement variability and comparability**

**Response:** Thank you for your important observation. We added new paragraphs discussing: differences between the 13-item and 30-item scales.

# **Response to Reviewer 1**

## Abstract

**Comment #1:** **The abstract lacks contextualization with regional/global maternal health burden.**

**Response:** We appreciate your constructive comment. We accept and revised the abstract to include current evidence on maternal mortality and poor quality of maternity.

**Comment #2: Replace “prevalence” with “pooled mean PCMC score.”**

**Response:** We kindly thank your insightful feedback. We accept and revised the abstract, results, discussion, and figures to consistently use “pooled mean PCMC score” instead of “prevalence” when referring to the continuous outcome.

**Comment #3: Add one line highlighting public health significance.**

**Response:** We thank you dear reviewer for the constructive comment, we accept and added the following sentence to the abstract conclusion: “Improving PCMC may contribute to increased institutional delivery, greater service utilization, and reductions in preventable maternal morbidity and mortality.”

## Introduction

**Comment #1: Add recent epidemiological data (e.g., recent maternal mortality, quality-of-care, and institutional delivery gaps data from East Africa).**

**Response:** We kindly thank you for the insightful comment. We accept and expanded the Introduction to include recent regional maternal mortality data, inequities in institutional delivery, and evidence that poor maternity care experiences reduce future healthcare-seeking.

**Comment #2: Add supporting evidence for reduced PCMC during nighttime delivery.**

**Response:** We thank you for insightful comment and we incorporated the suggested explanation and cited the recommended study. The Discussion now states: “Possible explanations include the limited number of care providers, the impact of physical weariness, workload, and weak supervision at night, which may reduce the focus on PCMC. Similar time-of-day variations in healthcare quality and service utilization have been reported in other public health contexts.”

**Comment #3: Strengthen the research gap as it is not strongly articulated.**

**Response:** Thank you in advance for your insightful feedback. We accept and modify accordingly. We added the following statement to strengthen the research gap: “Growing evidence regarding PCMC remains fragmented within individual studies, with no prior comprehensive synthesis examining the pooled PCMC estimate, subdomains, and predictors across East Africa, despite increasing institutional delivery in the region.”

## Methods

**Comment #1: Justify the restriction to studies conducted from 2017 onward.**

**Response:** Thank you for your valuable comment. We accept and modify accordingly. Studies published from 2017 onward were considered because the validated PCMC scale was first introduced and operationalized in 2017. Restricting the review to this period ensured comparability of the construct and avoided inclusion of studies that used non-standardized or conceptually different measures of maternity care experience. We also note that the earliest eligible PCMC studies in East Africa appeared after 2017.

**Comment #2: JBI quality assessment cutoff (>4.5/9) is arbitrary and not justified.**

**Response:** We sincerely thank you for your valuable comment. We accept and modify accordingly. The previous cutoff was insufficiently justified. We revised in the Methods section to explain that the JBI checklist contains nine items, with each item rated as “Yes”, “No”, “Unclear”, or Not applicable. While we report the score of individual items and total scores (out of 9) for transparency, JBI emphasizes that appraisal should be based on reviewer judgment rather than rigid threshold. Accordingly, for inclusion of studies we did not consider numerical cutoff; instead, we used the checklist to identify potential risk of bias (as low, moderate, and high risk of bias). This has now been added to the Results section stating: “All studies showed a low risk of bias”.

**Comment #3: Clarify outcome definition (mean vs. prevalence) as “prevalence” is conceptually incorrect.**

**Response:** We thank you for the constructive comment. We accept and clarified the outcome definition throughout the manuscript that the primary outcome was “the pooled mean PCMC score” or “the pooled estimate of PCMC” after rescaling all measures to a 0–100 scale.

**Comment #4: Justify use of DerSimonian–Laird despite high heterogeneity.**

**Response:** We thank the reviewer for this important observation regarding the use of the DerSimonian–Laird method in the presence of very high heterogeneity (I² ≈ 99%). We agree that such extreme heterogeneity warrants careful methodological justification and cautious interpretation. Our choice of a random-effects model using the DerSimonian–Laird estimator was made a priori, given the anticipated diversity across included studies, including differences in study populations, settings, and measurement tools.

However, we acknowledge its limitations, particularly the potential underestimation of between-study variance (τ²) under conditions of substantial heterogeneity. In response to this concern, we have strengthened the manuscript in several ways. First, we have explicitly justified the use of the random-effects framework in the Methods section. Second, we have conducted additional analyses to explore heterogeneity, including subgroup analyses and meta-regression using relevant study-level covariates. Third, we have revised the Results and Discussion sections to emphasize cautious interpretation of pooled estimates and to focus more on the consistency and direction of findings rather than the precise pooled magnitude.

**Comment #4: No meta-regression performed.**

**Response:** Thank you for the insightful comment. We have now conducted and reported meta-regression analyses.

## Results

**Comment #1: Extremely high heterogeneity makes pooled estimates potentially unreliable.**

**Response:** We thank you for your important observation. We revised the Results and Discussion section to explicitly state that the pooled estimates should be interpreted cautiously and that substantial between-study variability remained.

**Comment #2: Publication bias is present but interpretation is weak.**

**Response:** We appreciate your constructive comment. We strengthened the discussion of publication bias. We now state that the significant Egger’s test and asymmetry in the funnel plot suggest possible small-study effects or publication bias, which may have influenced the pooled estimate.

**Comment #3: Only Ethiopia and Kenya represented.**

**Response:** We appreciate the reviewer for this crucial observation. Despite a thorough and rigorous search across multiple databases and sources, eligible studies were identified only from Ethiopia and Kenya, likely reflecting gaps in the available literature rather than limitations of the search strategy. We have revised the manuscript to clarify the geographic scope of included studies and to avoid overgeneralization to the wider East African region, while highlighting that the consistency of findings across these settings offers meaningful evidence on person-centered maternity care in resource-constrained contexts. We further acknowledge the implications for external validity and emphasize that findings should be interpreted with caution when applied to other settings.

**Comment #4: “Prevalence” used incorrectly.**

**Response:** Thank you, we accept and corrected throughout the manuscript.

## Discussion

**Comment #1: Over interpretation of pooled estimates despite extreme hetrogeneity.**

**Response:** We appreciate your insightful observation. We accept and revised the Discussion and Conclusion section to use more cautious language and avoid implying that the pooled estimate precisely represents all East African countries.

**Comment #2: “Times higher/lower” is incorrect for β coefficients.**

**Response:** We sincerely appreciate this valuable observation. We accepted and corrected the interpretation of β coefficients throughout the manuscript. For example, instead of “Women who initiated ANC early had 3.5 times higher PCMC,” we now state: “Women who initiated antenatal care early had, on average, a 3.5-point higher PCMC score than those who initiated later.”

**Comment #3: Add discussion on measurement variability and cultural/system-level differences.**

**Response:** Thank you for your insightful observation. We added new paragraphs discussing:

- Differences between the 13-item and 30-item scales
- Differences in health systems, provider–patient interactions, and sociocultural expectations across countries

## Strengths and Limitations

**Comment #1: Underemphasized (missing) limitations.**

**Response:** Thank you for your important insight. Accept and expanded the Limitations section to explicitly include: very high heterogeneity, evidence of publication bias, limited regional representation, differences in PCMC measurement instruments, cross-sectional design of most included studies, inability to infer causality for predictors

## Conclusion

**Comment #1: Conclusions are overstated.**

**Response:** We revised the Conclusion to state:

“The pooled mean PCMC score suggests substantial gaps in person-centered maternity care in East Africa; however, findings should be interpreted cautiously because of high heterogeneity, publication bias, and limited regional representation.”

# Response to Reviewer 2

We sincerely thank the reviewer for the thoughtful and constructive feedback. We appreciate the positive recognition of the importance and timeliness of our study, as well as the strengths highlighted, including the focus on key PCMC subdomains, relevant predictors, adherence to PRISMA/MOOSE guidelines, and the large pooled sample size. We are grateful for the reviewer’s insightful suggestions to further strengthen the rigor, clarity, and overall impact of the manuscript. In response, we have carefully revised the manuscript to address all the points raised, as detailed below.

**Comment #1: Interpretation of Heterogeneity**

**Response:** We sincerely appreciate for your insightful comment. We agree and have added a more explicit discussion of what the very high I² implies. We now emphasize that heterogeneity likely reflects true differences between settings and that pooled results should be interpreted cautiously.

**Comment #2: Measurement Variability and Scale Harmonization**

**Response:** we appreciate for your important observation. We accept and added:

- A subgroup analysis by PCMC scale type (13-item and 30-item)
- Meta-regression including scale type
- Expanded discussion regarding the conceptual differences between the 13-item and 30-item scales
- A recommendation for future studies to use standardized PCMC instruments across settings

**Comment #3: Deeper interpretation of communication/autonomy and nighttime delivery**

**Response:** We thank you the reviewer for the valuable comment. We accept and expanded the Discussion to emphasize that low communication and autonomy scores may reflect:

- Provider–patient power imbalances
- Limited opportunity for shared decision-making
- High workload and insufficient training in interpersonal care

We also reframed nighttime delivery as a form of temporal inequity in care quality and discuss how staffing patterns, fatigue, and supervision may contribute.

**Comment #4: Strengthen the Recommendations**

**Response:** We sincerely appreciate for your insightful observation. We accept and revised the Recommendations section to make it more specific and actionable. We now recommend:

- Communication-skills and shared decision-making training for maternity care providers
- Simulation-based respectful maternity care training
- Inclusion of PCMC indicators within national maternal health quality monitoring systems
- Rural-targeted interventions, including community engagement and task-sharing approaches
- Implementation science research and intervention trials to improve communication and autonomy

**Comment #5: Abstract and Introduction Clarity**

**Response:** Thank you for your valuable comment. We accept and streamlined the abstract, reduced unnecessary methodological detail, and strengthened the introduction to clarify the evidence gap and novelty of the review.

Comment 6: Minor clarity and language edits

**Response:** Thank you for important observation. We accept and carefully edited for grammar, clarity, and consistency. We also corrected inaccurate statements such as references to studies “after 2025.”

# Response to Journal Data Availability Requirements

We have prepared and exhaustively uploaded all dataset and supplementary files including:

- S1 supporting information. PRISMA 2020 checklist.
- S2 supporting information. Full search strategies for databases.
- S3 supporting information. Studies identified in the literature search and screened for eligibility.
- S4 supporting information. Full extracted dataset for all studies.
  - Study identifiers
  - Extracted means, standard deviations, and sample sizes
  - Rescaled scores
  - Extractor names and extraction dates
  - Eligibility confirmation
- S5 supporting information. JBI quality assessment.

Figure files were processed using the PLOS NAAS tool and uploaded as NAAS-corrected TIF files (e.g., Figure1.tif). In accordance with PLOS style, figures are cited in the manuscript as “Fig 1,” while the corresponding uploaded file names use the format “Figure1.tif”.
