# Supplementary material for: Prevalence of person-centered maternity care during childbirth and its predictors among women who gave birth in health facilities of East Africa: A systematic review and meta-analysis
Source: PLOS Glob Public Health. 2026 Sep 29;6(9):e0005909. doi: 10.1371/journal.pgph.0005909 (PMC13623155; doi:10.1371/journal.pgph.0005909)
Supplement: Peer review history

# Peer review: Decision Letter 0

**DOI:** 10.1371/journal.pgph.0005909.r001

**Authors:** Ifunanya Agu

7 Apr 2026

PGPH-D-26-00155

Prevalence of person-centered maternity care during childbirth and its predictors among women who gave birth in health facilities of East Africa: a systematic review and meta-analysis

PLOS Global Public Health

Dear Dr. Admasu,

Thank you for submitting your manuscript to PLOS Global Public Health. After careful consideration, we feel that it has merit but does not fully meet PLOS Global Public Health’s publication criteria as it currently stands. Therefore, we invite you to submit a revised version of the manuscript that addresses the points raised during the review process.

Please submit your revised manuscript by 21st April 2026. If you will need more time than this to complete your revisions, please reply to this message or contact the journal office at globalpubhealth@plos.org. When you're ready to submit your revision, log on to https://www.editorialmanager.com/pgph/ and select the 'Submissions Needing Revision' folder to locate your manuscript file.

Please include the following items when submitting your revised manuscript:

- A letter that responds to each point raised by the editor and reviewer(s). You should upload this letter as a separate file labeled 'Response to Reviewers'.
- A marked-up copy of your manuscript that highlights changes made to the original version. You should upload this as a separate file labeled 'Revised Manuscript with Track Changes'.
- An unmarked version of your revised paper without tracked changes. You should upload this as a separate file labeled 'Manuscript'.

Guidelines for resubmitting your figure files are available below the reviewer comments at the end of this letter.

As the corresponding author, your ORCID iD is verified in the submission system and will appear in the published article. PLOS supports the use of ORCID, and we encourage all coauthors to register for an ORCID iD and use it as well. Please encourage your coauthors to verify their ORCID iD within the submission system before final acceptance, as unverified ORCID iDs will not appear in the published article. *Only*  the individual author can complete the verification step; PLOS staff *cannot*  verify ORCID iDs on behalf of authors.

We look forward to receiving your revised manuscript.

Kind regards,

Ifunanya Clara Agu

Academic Editor

PLOS Global Public Health

**Journal Requirements:**

1.  We note that your Data Availability Statement is currently as follows: The datasets used and/or analyzed during the current study are included within the manuscript.

Please confirm at this time whether or not your submission contains all raw data required to replicate the results of your study. Authors must share the “minimal data set” for their submission. PLOS defines the minimal data set to consist of the data required to replicate all study findings reported in the article, as well as related metadata and methods (https://journals.plos.org/plosone/s/data-availability#loc-minimal-data-set-definition).

For example, authors should submit the following data:

- The values behind the means, standard deviations and other measures reported;

- The values used to build graphs;

- The points extracted from images for analysis.

Authors do not need to submit their entire data set if only a portion of the data was used in the reported study.

If your submission does not contain these data, please either upload them as Supporting Information files or deposit them to a stable, public repository and provide us with the relevant URLs, DOIs, or accession numbers. For a list of recommended repositories, please see https://journals.plos.org/plosone/s/recommended-repositories.

If there are ethical or legal restrictions on sharing a de-identified data set, please explain them in detail (e.g., data contain potentially sensitive information, data are owned by a third-party organization, etc.) and who has imposed them (e.g., an ethics committee). Please also provide contact information for a data access committee, ethics committee, or other institutional body to which data requests may be sent. If data are owned by a third party, please indicate how others may request data access.

2. As required by our policy on Data Availability, please ensure your manuscript or supplementary information includes the following:

A numbered table of all studies identified in the literature search, including those that were excluded from the analyses.

For every excluded study, the table should list the reason(s) for exclusion.

If any of the included studies are unpublished, include a link (URL) to the primary source or detailed information about how the content can be accessed.

A table of all data extracted from the primary research sources for the systematic review and/or meta-analysis. The table must include the following information for each study:

Name of data extractors and date of data extraction

Confirmation that the study was eligible to be included in the review.

All data extracted from each study for the reported systematic review and/or meta-analysis that would be needed to replicate your analyses.

If data or supporting information were obtained from another source (e.g. correspondence with the author of the original research article), please provide the source of data and dates on which the data/information were obtained by your research group.

If applicable for your analysis, a table showing the completed risk of bias and quality/certainty assessments for each study or outcome.  Please ensure this is provided for each domain or parameter assessed. For example, if you used the Cochrane risk-of-bias tool for randomized trials, provide answers to each of the signalling questions for each study. If you used GRADE to assess certainty of evidence, provide judgements about each of the quality of evidence factor. This should be provided for each outcome.

An explanation of how missing data were handled.

This information can be included in the main text, supplementary information, or relevant data repository. Please note that providing these underlying data is a requirement for publication in this journal, and if these data are not provided your manuscript might be rejected.

If the reviewer comments include a recommendation to cite specific previously published works, please review and evaluate these publications to determine whether they are relevant and should be cited. There is no requirement to cite these works unless the editor has indicated otherwise.

**Additional Editor Comments (if provided):**

Dear Dr. Admasu,

Your manuscript has now been evaluated by two independent reviewers with relevant expertise. Both reviewers agree that the topic is important and timely, and that the study has the potential to contribute meaningfully to the evidence base on person-centered maternity care in East Africa. The use of a systematic review and meta-analysis framework, along with adherence to PRISMA/MOOSE guidelines, is also recognized as a strength.

However, the reviewers have raised several substantive concerns regarding methodological rigor, clarity of reporting, and interpretation of findings. In light of these comments, I invite you to revise your manuscript before it can be considered further for publication.

Some of the most critical issues that need to be addressed are below:

1. Interpretation of heterogeneity and validity of pooled estimates

The reported heterogeneity is extremely high (I² ≈ 99%), which raises important concerns regarding the appropriateness and interpretability of pooled estimates. The manuscript should provide a clearer and more cautious interpretation of these findings, including a deeper exploration of potential sources of heterogeneity and their implications for validity.

2. Methodological transparency and rigor

Several aspects of the methods require clarification or strengthening, including:

Justification for restricting included studies to those published from 2017 onward

Justification of the JBI quality assessment cutoff

Consideration of additional analyses (e.g., meta-regression) to explore heterogeneity

More explicit reporting and justification of statistical approaches used

3. Measurement variability and comparability

The inclusion of both 13-item and 30-item PCMC scales introduces comparability challenges. While rescaling was performed, the manuscript should provide a more critical discussion of whether these tools measure equivalent constructs, and more clearly acknowledge this limitation.

The conclusions should be moderated to reflect the limitations of the analysis. Additionally, recommendations could be made more specific and actionable, particularly in terms of policy and practice implications. Please provide a detailed, point-by-point response to each reviewer comment when submitting your revised manuscript.

We would be happy to consider a revised version that adequately addresses these concerns. Please note that revision does not guarantee acceptance, and the manuscript may be returned to the reviewers for further evaluation.

Thank you for your interest in PLOS Global Public Health. We look forward to receiving your revised manuscript on 21st April 2026.

Sincerely,

Ifunanya Agu

Academic Editor

PLOS Global Public Health

Reviewers' comments:

Reviewer's Responses to Questions

**Comments to the Author**

1. Does this manuscript meet PLOS Global Public Health’s publication criteria?

Reviewer #1: Yes

Reviewer #2: Yes

**********

2. Has the statistical analysis been performed appropriately and rigorously?

Reviewer #1: Yes

Reviewer #2: Yes

**********

3. Have the authors made all data underlying the findings in their manuscript fully available (please refer to the Data Availability Statement at the start of the manuscript PDF file)?

The PLOS Data policy

Reviewer #1: Yes

Reviewer #2: Yes

**********

4. Is the manuscript presented in an intelligible fashion and written in standard English?

Reviewer #1: Yes

Reviewer #2: Yes

**********

**Reviewer #1:** Abstract

The abstract lacks contextualization with regional/global maternal health burden, reducing the perceived importance of the study.

The term “prevalence” is methodologically inconsistent with reporting pooled mean scores (0–100 scale). Replace “prevalence” with “pooled mean PCMC score” where appropriate.

Add one line highlighting the public health significance (e.g., maternal mortality or service utilization impact).

Introduction

Lacks recent epidemiological data (e.g., maternal mortality, institutional delivery gaps) to justify urgency.

The discussion on reduced PCMC during nighttime delivery lacks supporting evidence and should be strengthened with relevant literature demonstrating time-of-day variations in healthcare quality and outcomes. Incorporating such evidence will improve the scientific rigor and contextual validity of the proposed explanation. Add below phrase with recent temporal study..

“Possible explanations include the limited number of care providers, the impact of physical weariness, workload, and weak supervision at night, which may reduce the focus on PCMC; similar time-of-day variations in healthcare outcomes and service utilization patterns have been reported in other public health contexts [Sheikh et al.,2024, https://www.sciencedirect.com/science/article/pii/S294983412400028X ].”

The research gap is not strongly articulated.

Authors can insert:

“The introduction lacks incorporation of recent maternal mortality and quality-of-care data from East Africa, which is essential to justify the public health significance and urgency of evaluating person-centered maternity care.”

Methods

Search strategy limitation: Restricting studies from 2017 onward is not justified. Justify year restriction.

Quality assessment: Cutoff (>4.5/9) for JBI is arbitrary and not justified.

Referring to pooled mean scores as “prevalence” is conceptually incorrect. Clarify outcome definition (mean vs prevalence).

Use of DerSimonian–Laird method without justification despite very high heterogeneity (I² ~99%).

No meta-regression performed despite extreme heterogeneity. Consider meta-regression for heterogeneity.

Results

Extremely high heterogeneity (I² = 99.7%):

Pooled estimates may not be reliable.

Publication bias present: Egger’s test significant, yet interpretation is weak. Add cautious interpretation due to heterogeneity and bias.

Only Ethiopia and Kenya → limits generalizability to “East Africa”.

“Prevalence” used instead of “mean score”.

Discussion

Overinterpretation of pooled estimates despite extreme heterogeneity.

Inconsistent wording: “times lower/higher” used for β coefficients (incorrect interpretation). Interpret β correctly as unit change, not multiplicative effect.

Limited critical discussion on: Measurement variability (13 vs 30 item scale) and Cultural/system-level differences. Add discussion on measurement tool variability and its impact.

Strengths and Limitations

Limitations (missing or underemphasized): Very high heterogeneity. Publication bias. Limited country representation. Cross-sectional nature of included studies.

Conclusion

Slightly overstated conclusions given methodological limitations.

Needs more cautious wording. Add:

“Findings should be interpreted with caution due to high heterogeneity and limited regional representation.”

**Reviewer #2:**  This manuscript addresses an important and timely topic of person-centered maternity care in East Africa using a systematic review and meta-analysis approach. The focus on subdomains (dignity/respect, communication/autonomy, and supportive care) and predictors such as timing of delivery and ANC initiation adds meaningful clinical and policy relevance. The adherence to PRISMA/MOOSE guidelines and the relatively large pooled sample are notable strengths.

However, several areas would benefit from further refinement to strengthen the rigor, clarity, and impact of the manuscript:

1. Interpretation of Heterogeneity

The reported heterogeneity is extremely high (I² ~99%), which raises concerns about the appropriateness and interpretability of pooled estimates. While subgroup analyses are presented, the manuscript would benefit from a more explicit discussion of what this level of heterogeneity implies for validity. Consider elaborating on potential sources and clearly emphasizing cautious interpretation of pooled findings.

2. Measurement Variability and Scale Harmonization

The inclusion of both 13-item and 30-item PCMC scales, while pragmatic, introduces comparability challenges. Although rescaling was performed, further discussion is needed on whether these tools capture equivalent constructs. A sensitivity analysis stratified by scale (if not already emphasized) and a stronger acknowledgment of this limitation would improve methodological transparency. Additionally, the manuscript could highlight the need for standardized PCMC measurement across settings.

3. Depth of Interpretation (Discussion Section)

The discussion is generally well-structured but could be strengthened by moving beyond descriptive comparisons. For example:bThe consistently lower scores in communication and autonomy represent a critical systems-level gap and warrant deeper exploration (e.g., provider–patient power dynamics, time constraints, training gaps).

The finding of poorer PCMC during nighttime delivery is particularly compelling and could be framed as a form of temporal inequity in care quality, rather than solely a staffing issue.

4. Strengthening the Recommendations

The current recommendations (training, resource allocation, policy alignment) are appropriate but somewhat general. Consider making them more specific and actionable:

Specify the type of provider training (e.g., communication skills, shared decision-making, simulation-based learning).

Propose integration of PCMC indicators into national quality monitoring frameworks or facility-level accountability systems.bSuggest targeted interventions for rural disparities (e.g., task-shifting models, community engagement strategies).bHighlight the need for implementation science research and intervention trials, particularly focused on improving communication and autonomy.

5. Abstract and Introduction Clarity

The abstract could be streamlined by reducing methodological detail and incorporating brief interpretation of findings. In the introduction, the literature gap could be more explicitly articulated to better emphasize the novelty of this synthesis.

6. Minor Clarity and Language Edits

There are occasional grammatical inconsistencies and phrasing issues that would benefit from careful editing for clarity and conciseness. Additionally, some statements (e.g., regarding inclusion of studies “after 2025”) should be revised for accuracy.

**********

what does this mean?). If published, this will include your full peer review and any attached files.

**Do you want your identity to be public for this peer review?**  If you choose “no”, your identity will remain anonymous but your review may still be made public.

For information about this choice, including consent withdrawal, please see our Privacy Policy

Reviewer #1: No

Reviewer #2: No

**********

[NOTE: If reviewer comments were submitted as an attachment file, they will be attached to this email and accessible via the submission site. Please log into your account, locate the manuscript record, and check for the action link "View Attachments". If this link does not appear, there are no attachment files.]

**Figure Resubmissions:**

# Author comment: Author response to Decision Letter 1

**DOI:** 10.1371/journal.pgph.0005909.r002

26 May 2026

Attachment Submitted filename: Response to Reviewers.docx

# Peer review: Decision Letter 1

**DOI:** 10.1371/journal.pgph.0005909.r003

**Authors:** Angela Baschieri

9 Sep 2026

Prevalence of person-centered maternity care during childbirth and its predictors among women who gave birth in health facilities of East Africa: a systematic review and meta-analysis

PGPH-D-26-00155R1

Dear Mr Admasu,

We are pleased to inform you that your manuscript 'Prevalence of person-centered maternity care during childbirth and its predictors among women who gave birth in health facilities of East Africa: a systematic review and meta-analysis' has been provisionally accepted for publication in PLOS Global Public Health.

Before your manuscript can be formally accepted you will need to complete some formatting changes, which you will receive in a follow up email. A member of our team will be in touch with a set of requests.

Please note that your manuscript will not be scheduled for publication until you have made the required changes, so a swift response is appreciated.

IMPORTANT: The editorial review process is now complete. PLOS will only permit corrections to spelling, formatting or significant scientific errors from this point onwards. Requests for major changes, or any which affect the scientific understanding of your work, will cause delays to the publication date of your manuscript.

If your institution or institutions have a press office, please notify them about your upcoming paper to help maximize its impact. If they'll be preparing press materials, please inform our press team as soon as possible -- no later than 48 hours after receiving the formal acceptance. Your manuscript will remain under strict press embargo until 2 pm Eastern Time on the date of publication. For more information, please contact globalpubhealth@plos.org.

Thank you again for supporting Open Access publishing; we are looking forward to publishing your work in PLOS Global Public Health.

Best regards,

Angela Baschieri, PhD

Academic Editor

PLOS Global Public Health

***********************************************************

Thanks for addressing the reviewers comments, we are pleased to accept the paper for publication.

Reviewer Comments (if any, and for reference):

Reviewer's Responses to Questions

**Comments to the Author**

Reviewer #1: All comments have been addressed

Reviewer #3: All comments have been addressed

**********

publication criteria?

Reviewer #1: Yes

Reviewer #3: Yes

**********

3. Has the statistical analysis been performed appropriately and rigorously?

Reviewer #1: Yes

Reviewer #3: Yes

**********

4. Have the authors made all data underlying the findings in their manuscript fully available (please refer to the Data Availability Statement at the start of the manuscript PDF file)?

The PLOS Data policy

Reviewer #1: Yes

Reviewer #3: Yes

**********

5. Is the manuscript presented in an intelligible fashion and written in standard English?

Reviewer #1: Yes

Reviewer #3: Yes

**********

Reviewer #1: The authors have adequately addressed all reviewer comments and revised the manuscript accordingly. The revised version is scientifically sound, well-presented, and is now suitable for acceptance for publication.

Reviewer #3: There are few repetitions in the methods and results

**********

what does this mean?). If published, this will include your full peer review and any attached files.

**Do you want your identity to be public for this peer review?**  If you choose “no”, your identity will remain anonymous but your review may still be made public.

For information about this choice, including consent withdrawal, please see our Privacy Policy

Reviewer #1: No

Reviewer #3: No

**********
